# Pump-probe spectroscopy of chiral vibrational dynamics

**DOI:** 10.1126/sciadv.ade0311

**Published:** 2022-12-07

**Authors:** Denis S. Tikhonov, Alexander Blech, Monika Leibscher, Loren Greenman, Melanie Schnell, Christiane P. Koch

**Affiliations:** ^1^ Deutsches Elektronen-Synchrotron DESY, Notkestr. 85, 22607 Hamburg, Germany.; ^2^ Institute of Physical Chemistry, Christian-Albrechts-Universität zu Kiel, Max-Eyth-Str. 1, 24118 Kiel, Germany.; ^3^ Dahlem Center for Complex Quantum Systems and Fachbereich Physik, Freie Universität Berlin, Arnimallee 14, 14195 Berlin, Germany.; ^4^ Department of Physics, Kansas State University, 116 Cardwell Hall, 1228 N. 17th St., Manhattan, KS 66506-2601, USA.

## Abstract

A planar molecule may become chiral upon excitation of an out-of-plane vibration, changing its handedness during half a vibrational period. When exciting such a vibration in an ensemble of randomly oriented molecules with an infrared laser, half of the molecules will undergo the vibration phase-shifted by π compared to the other half, and no net chiral signal is observed. This symmetry can be broken by exciting the vibrational motion with a Raman transition in the presence of a static electric field. Subsequent ionization of the vibrating molecules by an extreme ultraviolet pulse probes the time-dependent net handedness via the photoelectron circular dichroism. Our proposal for pump-probe spectroscopy of molecular chirality, based on quantum-chemical theory and discussed for the example of the carbonyl chlorofluoride molecule, is feasible with current experimental technology.

## Teaser

Planar molecules can be made chiral with electric fields only, and a net chiral signature can be recorded upon ionization.

## INTRODUCTION

Polyatomic molecules are often chiral, i.e., they exist in a left-handed and a right-handed version that cannot be superimposed by rotations and translations. The two conformers differ markedly in their chemical and biological behavior including a preference of handedness in amino acids and sugars in living organisms that is not yet understood (*1*). While handedness refers to the nuclear scaffold, molecular chirality is governed by the electronic structure of the molecule — left-handed molecules are separated from right-handed ones by a potential barrier. The height of this barrier determines the time scale over which the two conformers interconvert. Molecular chirality would be static only for infinitely high potential barriers. In real molecules, the lifetimes of a molecule as a specific enantiomer span a huge range (*2*). The barrier can be overcome by tunneling, vibrational excitation, or excitation to an achiral (and thus barrierless) electronically excited state. The corresponding interconversion dynamics may be exploited in asymmetric photochemistry (*3*). It is also at the core of proposals for coherent spectroscopies, for example, to measure the effect of parity violation (*2*, *4*) or to coherently control enantiomeric purification (*5*, *6*, *7*).

None of these proposals has been realized in an experiment as of yet. This may be attributed to two main factors — (i) the complexity of the process involving chiral-to-achiral excitation, dynamics in the achiral state, and achiral-to-chiral deexcitation, all of which have to be driven selectively, and the choice of suitable molecules (*8*); and (ii) the lack of diagnostic tools for chirality-changing dynamics, in particular when the molecules are randomly oriented. The latter has been remedied by the advent of chiral vector observables that require only electric dipole transitions (*9*), and among these, photoelectron circular dichroism (PECD) (*10*–*12*) is particularly versatile and suitable to monitor ultrafast dynamics (*13*–*15*). The complexity of studying interconversion dynamics can be reduced by separating the transitions between chiral and achiral states (*15*–*17*) and intermediate dynamics. However, fragmentation (*16*) and dissociation (*15*, *17*) do not provide a viable route toward studying interconversion because the molecules are ripped apart. A more benign approach depositing less energy into the molecules is needed to probe interconversion dynamics, for example, using vibrational excitation.

**Fig. 1.**
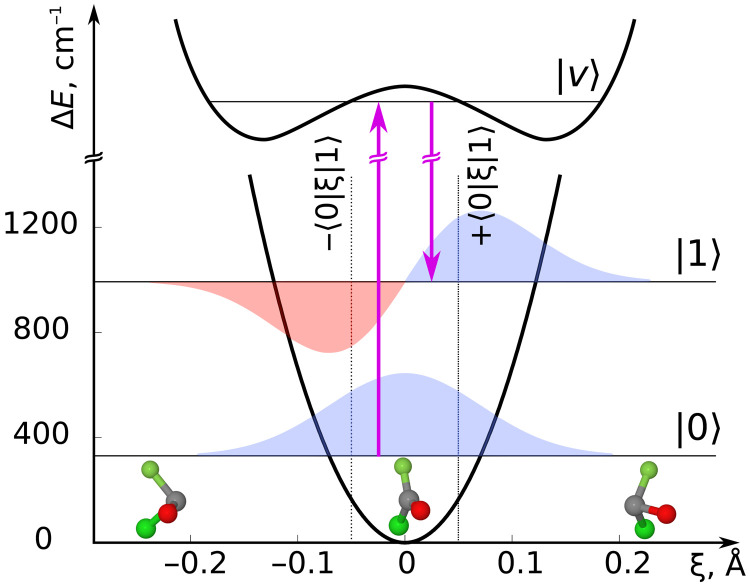
Creation of a chiral vibrational wave packet in a planar molecule. Raman excitation (magenta arrows) of a superposition of the two lowest levels of the OOP vibration via a prochiral electronically excited state with vibrational level ∣*v*〉. In our example of carbonyl chlorofluoride (COFCl), ξ is the distance of the carbon atom from the O-F-Cl plane, and the vertical dotted lines at ± 〈 0 ∣ ξ ∣ 1〉 indicate the maximal average displacement of the vibrational wave packet.

Here, we suggest, based on state-of-the-art quantum-chemical calculations, to induce and probe chiral vibrational motion: achiral molecules have a symmetry plane in their equilibrium geometry but oscillate between left-handed and right-handed configurations during out-of-plane (OOP) vibrational motion (cf. Fig. 1). While the eigenstates are either symmetric or antisymmetric with respect to the symmetry plane and thus racemic, coherent superposition states are chiral (*18*–*20*) and change their handedness as a function of time. This can be probed, for example, with ultrafast PECD (*12*–*15*). For a single molecule, the chirality is largest when probing at the maximal average OOP displacement, indicated by the vertical dotted lines in Fig. 1 for the simplest superposition involving only the ground and first excited level. However, for an ensemble of molecules with random orientations, the chirality will average out, yielding zero net enantiomeric excess. We show that Raman excitation in the presence of a static electric field yields a nonvanishing time-dependent enantiomeric excess, reflecting chiral vibrational motion in an ensemble of randomly oriented achiral molecules. We calculate the time-dependent PECD for the example of planar carbonyl chlorofluoride (COFCl) and discuss the requirements of a corresponding pump-probe study.

## RESULTS

Focusing on the simplest superposition, ∣ψ(*t*)〉 = *c*_0_∣0〉 + $c_{1}e^{-i(E_{1}-E_{0})t/h}|1\rangle$, the largest OOP displacement is obtained for equal weights. Assuming the molecule initially in its ground vibrational state, such a superposition can, in principle, be prepared by an infrared (IR) pulse or via ultraviolet (UV) Raman excitation. However, IR excitation does not produce a nonzero enantiomeric excess, as shown in the following. In both cases, the laser-molecule interaction depends on the orientation of the molecular axes relative to the laser polarization

$${\hat{H}}_{\mathrm{int}}=-E(t)\;R(\varphi ,\theta ,\chi )\;\hat{\mu }$$

where $\hat{\mu }$ is the electric dipole moment in the molecular frame, **E**(*t*) is the electric field in the laboratory frame, and R is the rotation matrix connecting the two coordinate systems, dependent on the Euler angles φ, θ, and χ (*21*).

**Fig. 2.**
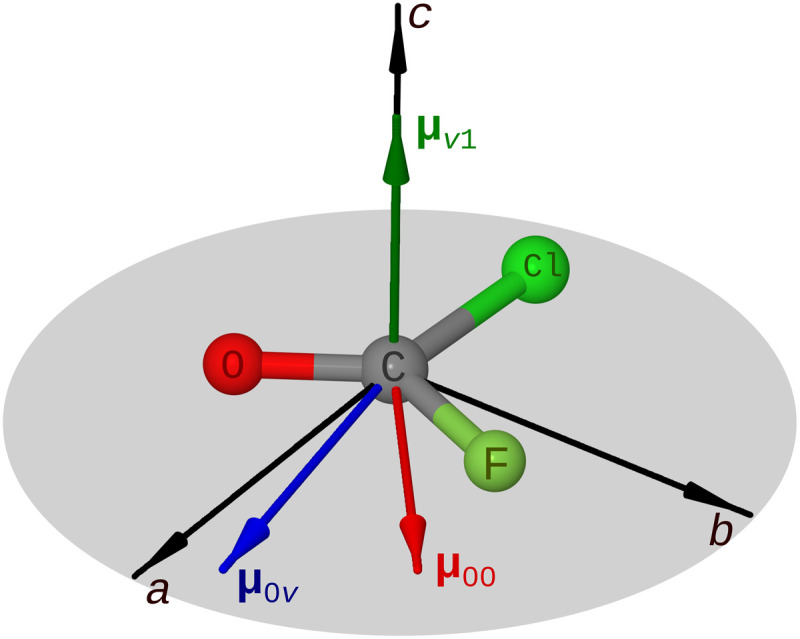
Molecular geometry together with the dipole moments relevant for creating a chiral vibrational wave packet. Molecular frame representation of the permanent (**μ**_00_) and transition (**μ**_0*v*_ and **μ**_*v*1_) dipole moments of COFCl for a symmetric vibrational level ∣*v*〉.

The net enantiomeric excess 〈 〈 ξ 〉 〉 (*t*) in a gas-phase sample is obtained by averaging the mean OOP displacement, 〈 ξ 〉 (*t*) = 〈 ψ(*t*) ∣ ξ ∣ ψ(*t*)〉, over all molecular orientations (*22*). For an isotropic ensemble, the net enantiomeric excess is zero after rotational averaging for both IR and Raman excitation, because at least three mutually orthogonal fields are required to break the spatial symmetry (see section SI.B in the Supplementary Materials for a detailed explanation) (*23*). One option to fulfill this requirement is to apply a weak static electric field in conjunction with a circularly polarized Raman pulse. The direction of the relevant dipole moments, determined by the symmetry of the corresponding wave functions, is schematically shown in Fig. 2. In the presence of **E**_static_ and assuming the molecules to be in thermal (rotational) equilibrium at temperature *T*, the distribution of Euler angles is given by the Boltzmann factor *p*(φ, θ, χ) = exp[**E**_static_R(φ, θ, χ)**μ**_00_/(*k*_B_*T*)] with $\mu_{00}=\langle 0|\hat{\mu }|0\rangle$ the dipole moment of the molecule in the ground state, which lies in the molecular plane (see Fig. 2). Realistic field strengths are small on the scale of thermal excitations, and the Boltzmann factor can be approximated

$$p(\varphi ,\theta ,\chi )\approx 1+E_{\mathrm{static}}R(\varphi ,\theta ,\chi )\mu_{00}/(k_{B}T)$$ (1)

We use perturbation theory (PT) in the following, which nicely captures the essence of the suggested excitation scheme. A Raman transition requires PT to second order and involves the transition dipole moments $\mu_{0v}=\langle 0|\hat{\mu }|v\rangle$ and $\mu_{v1}=\langle v|\hat{\mu }|1\rangle$, where *v* can be a symmetric or antisymmetric vibrational level in an electronically excited state (see Fig. 1). Averaging a second-order process over the rotational distribution Eq. 1 results in triple products of both electric fields and molecular dipole moments (see section SI.C in the Supplementary Materials),

$$\begin{array}{rl} \left\langle \langle \xi \rangle \right\rangle & (t)\propto \langle 0|\xi |1\rangle \sin(\omega_{01}t)\;\frac{\tau^{2}}{T}\;{\left[1+\mathrm{erf}\;\left(\frac{t}{\tau }\right)\right]}^{2}\\ & \times [E_{\mathrm{static}}\cdot (E_{L1}\times E_{L2})][\mu_{00}\cdot (\mu_{0v}\times \mu_{v1})] \end{array}$$ (2)

where we have assumed a circularly polarized Gaussian pulse with duration τ and polarization directions **E**_L1/2_. Here, *ω*_01_ denotes the OOP vibrational frequency, and the erf(*x*) is the error function, which becomes approximately constant for *t* > 1.5τ. As for many enantio-sensitive observables (*9*), this expression separates into products of the fields in the laboratory frame and molecular quantities in the molecular frame, which independently determine the amount of enantiomeric excess. The triple product of the fields is maximized by choosing all three fields orthogonal to each other, e.g., a static field along the *z* direction and a Raman pulse circularly polarized in the *xy* plane. If the intermediate state∣*v*〉 is totally symmetric (*A*′), then only the molecular frame components $\mu_{0v}^{a}$, $\mu_{0v}^{b}$, and $\mu_{v1}^{c}$ can be nonzero, i.e., **μ**_0*v*_ lies in the *ab* plane, and **μ**_*v*1_ is perpendicular to it (see Fig. 2). In general, **μ**_0*v*_ does not have the same direction as **μ**_00_, and thus, the triple product [**μ**_00_ · (**μ**_0*v*_ × **μ**_*v*1_)] is nonzero. The same argument holds for antisymmetric (*A*′′) intermediate states, where the elements $\mu_{0v}^{c}$, $\mu_{v1}^{a}$, and $\mu_{v1}^{b}$ do not vanish by symmetry. Raman excitation in the presence of a weak static electric field can thus induce chiral wave packets with nonvanishing time-dependent enantiomeric excess in an ensemble of randomly oriented planar molecules. Excitation of the vibrational superposition with a circularly polarized IR pulse in the presence of a static electric field does not result in a net chiral signature. Within first-order PT, rotational averaging results in (**E**_static_ · **E**_L_)(**μ**_00_ · **μ**_01_), which vanishes because **μ**_00_ and **μ**_01_ are orthogonal because of molecular symmetry (details are found in section SI.D in the Supplementary Materials).

We now show that the nonvanishing time-dependent enantiomeric excess created by UV Raman excitation in the presence of a static field can be probed by a time-delayed ionizing pulse via PECD. PECD refers to the forward-backward asymmetry of photoelectron angular distributions (PADs) upon ionization of chiral molecules with left and right circularly polarized (LCP/RCP) light (*10*). Denoting the laboratory-frame photoelectron momentum by **k** and following the convention in (*24*), it is given by the normalized dichroic difference

$$\mathrm{PECD}(k)=\frac{{\mathrm{PAD}}^{\mathrm{LCP}}(k)-{\mathrm{PAD}}^{\mathrm{RCP}}(k)}{[{\mathrm{PAD}}^{\mathrm{LCP}}(k)+{\mathrm{PAD}}^{\mathrm{RCP}}(k)]/2}$$ (3)

For an achiral molecule, PECD is zero, while for a chiral molecule, PECD changes sign if one enantiomer is exchanged by the other. If a chiral wave packet is excited in an achiral molecule, then an oscillation of the PECD with the vibrational period is expected, provided that the probe pulse is sufficiently short.

**Fig. 3.**
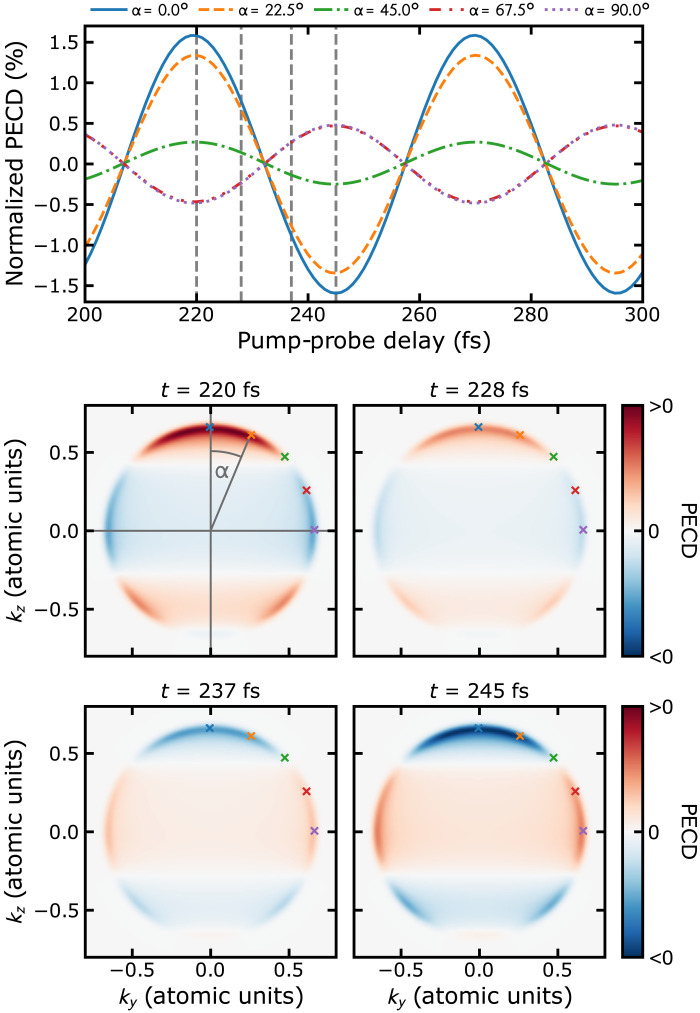
Pump-probe spectroscopy of light-induced chirality. Maximum PECD signal at various polar angles normalized to the mean photoelectron intensity (top). Differential photoelectron spectra in the velocity map image (VMI) *yz* plane for the four times indicated by dashed vertical lines in the top panel. The crosses correspond to the polar angles shown in the top panel.

The top panel of Fig. 3 displays PECD in a sample of COFCl molecules at a rotational temperature of 1 K as a function of the time delay *t* between the Raman excitation and the extreme ultraviolet (XUV) probe pulse. Velocity map images (VMIs) of PECD, i.e., the projection of Eq. 3 onto the *yz* plane, are shown for four time delays in the bottom panels of Fig. 3. The photoelectron asymmetry is presented in terms of the maximum dichroic difference along individual polar angles on the VMI detection plane, normalized relative to the mean photoelectron intensity. Expressing PECD in terms of a single number by integrating over the forward and backward hemispheres as commonly done in experiments (*25*–*27*) is not possible here (see section SII.C in the Supplementary Materials). The pump pulse is assumed to be Gaussian with a peak intensity of 10^13^ W/cm^2^ and 150 fs full width at half maximum (FWHM), circularly polarized in the *xy* plane, and resonant to excited-state vibrational levels *v* = 20, 21 (because PECD is a differential measurement, contributions from other transitions that might be driven by the Raman pulse cancel out). The probe pulse, with 5 fs FWHM and applied after a time delay *t*, is also circularly polarized in the *xy* plane. Its central frequency of 16.98 eV is chosen such that PECD is maximal. With these parameters, the energy dependence of the PAD is in good approximation given by the ionizing pulse spectrum (see section SII.C in the Supplementary Materials). The static electric field is taken along the *z* direction with a field strength of 5 × 10^5^ V/cm, which can be achieved, e.g., in modern Stark decelerators (*28*).

## DISCUSSION

The chiral pump-probe signal in Fig. 3 oscillates with a beat period of around 50 fs, as expected from the frequency of the OOP vibration. These oscillations confirm a net switch of handedness in the molecular ensemble on the time scale of the vibrational motion. Under the assumptions made above, the maximum PECD is of the order of 1.5%. This is an order of magnitude smaller than what has been observed in chiral molecules such as fenchone (*12*, *13*) but is well within the capabilities of current photoelectron detection (*27*). The strength of PECD could be increased by optimizing the probe pulse, e.g., by using a polarization-shaped pulse (*29*, *30*), which would maximize the asymmetry in the PAD. Alternatively, the chirality of the individual molecules could be increased by creating a vibrational superposition with larger maximal OOP displacement. While this has the chance to increase PECD, it may suffer from possible chiral zeros, because PECD can exhibit sign changes even if the handedness of the light and the molecule are kept fixed (*31*, *32*). In addition, it comes at the expense of suitably tailoring the Raman excitation. A third option would be to amend the static electric field by an additional nonresonant laser pulse to increase the degree of orientation (*33*).

For the prediction of Fig. 3 to be observable, the coherent vibrational dynamics should not be washed out by decoherence. Mechanisms with time scales that are potentially of the order of 1 ps or below are rotational dephasing arising from rovibrational energy differences and intramolecular vibrational energy redistribution (IVR). A full account is provided in section SIII in the Supplementary Materials. We find the time scale for rotational dephasing to exceed 1 ps already at 70 K, becoming even larger for the cold ensembles that we consider. IVR is due to anharmonic coupling between different vibrational modes. Using ab initio molecular dynamics methods at the B3LYP-D3/def2-SVP level of theory, we find the relevant time scale to be ∼3.5 ps, comparable to what has been found for the C─O stretch mode in hexafluoroacetone (*34*) and sufficiently large for the coherent pump-probe spectroscopy that we suggest.

Other candidate molecules besides COFCl include formic acid, which has been used in enantioselective fragmentation (*16*) but comes with a somewhat shorter vibrational period of 32 fs, and salts of the third main group such as AlCl_3_. Substitution with two electron-withdrawing groups (halogens or CF_3_) and one electron-donating (like CH_3_) would make them more polar while keeping the boiling point low (*35*–*37*), important for use in molecular beams. The latter facilitate low vibrational and rotational temperatures, increasing the effective orientation in the static electric field and reducing rotational dephasing.

To summarize, we have laid out the principles for studying chiral vibrational dynamics in a gas-phase ensemble of planar, i.e., achiral molecules. Nonzero enantiomeric excess will be obtained if the field configuration, used to create the chiral vibrational superposition, ensures the characteristic triple products in rotational averages (*9*). Such a configuration can be realized with a static electric field, such that the molecules are uniaxially oriented, and an ultrashort circularly polarized Raman pulse that creates the vibrational wave packet, ideally with its polarization plane perpendicular to the static field. The induced time-dependent chirality can be probed by PECD upon ionization with a sufficiently short, time-delayed, circularly polarized pulse. The chiral signals that we predict on the basis of quantum-chemical calculations for the test case of planar COFCl are readily observable, and a corresponding experiment is feasible with existing technical capabilities. Such studies of time-dependent chirality, following the change of handedness in the course of vibrational motion, would provide a stepping stone to lastly realize long-standing proposals for chiral molecules, including the measurement of parity violation (*2*, *4*) and enantiomeric purification with light (*5*–*7*).

## METHODS

The electronic properties of the neutral molecule, i.e., potentials and dipole moments as a function of the OOP mode ξ, are calculated at the B3LYP level of theory (*38*–*41*) with the cc-pVTZ basis set (*42*), making use of the Tamm-Dancoff approximation (*43*) for the excited state, using Orca 4 (*44*). The vibrational eigenstates and (transition) dipole moments **μ**_00_, **μ**_0*v*_, and **μ**_*v*1_ are obtained by numerically solving the vibrational Schrödinger equation using a discrete variable representation (*45*) for the ground and excited electronic state. The vibrational frequency of the electronic ground-state OOP mode and the UV absorption spectrum reproduce the available experimental data for COFCl (see section SII, A and B, in the Supplementary Materials for details) (*46*–*50*).

To describe ionization, the transition dipole moments for the bound-continuum transition are needed. The bound-state orbitals of COFCl required to this end have been calculated within the Hartree-Fock approximation using MOLPRO (*51*, *52*) with the aug-cc-pVTZ basis set (*53*) for a set of molecular configurations along the OOP coordinate. The continuum wave functions and ionization matrix elements have been obtained with ePolyScat (*54*, *55*) in the frozen-core static-exchange approximation following (*29*, *30*, *56*). Ionization is assumed to be instantaneous, keeping the molecular configuration fixed at its equilibrium structure except for the OOP vibration. The instantaneous PAD of the vibrating molecule is then obtained by averaging the ionization matrix elements over the OOP coordinate. The interaction with both pump and probe pulse is treated perturbatively, which is justified because by very small transition moments. PADs are calculated for different Euler angles and then averaged over the rotational distribution (1) (see also section SII.C in the Supplementary Materials).

The IVR lifetime was estimated using quantum-chemical molecular dynamics simulations done with Orca 4 (*44*) at the B3LYP-D3BJ/def2-SVP (*38*–*41*, *57*, *58*) level of theory. The initial coordinates of the nuclei were taken as displacements along the OOP coordinate ξ with all other modes in their equilibrium geometry, while the velocities were assigned using a Maxwell-Boltzmann distribution at a temperature of 300 K. More details are available in section SIII.C in the Supplementary Materials.

## Supplementary Materials

### This PDF file includes:

Sections I to VI Figs. S1 to S11 Tables S1 to S4 References
