# Supplementary material for: Pump-probe spectroscopy of chiral vibrational dynamics
Source: Sci Adv. 2022 Dec 7;8(49):eade0311. doi: 10.1126/sciadv.ade0311 (PMC9728962; doi:10.1126/sciadv.ade0311)
Supplement: Supplementary file 1 — Sections I to VI Figs. S1 to S11 Tables S1 to S4 References [file sciadv.ade0311_sm.pdf]

Science Advances
AAAS

Supplementary Materials for

Pump-probe spectroscopy of chiral vibrational dynamics

Denis S. Tikhonov *et al.*

Corresponding author: Melanie Schnell, melanie.schnell@desy.de;
Christiane P. Koch, christiane.koch@fu-berlin.de

Sci. Adv. **8**, eade0311 (2022)
DOI: 10.1126/sciadv.ade0311

This PDF file includes:

Sections SI to SVI
Figs. S1 to S11
Tables S1 to S4
References

I. GENERAL CONSIDERATIONS

A. Geometry of the envisioned experiment

The proposed experiment is sketched in Figure S1: Two oppositely charged electrodes (indicated by the red and green rings with "+" and "−" for the charges) create a static electric field with strength $\mathbf{E}_{\text{static}}$. A circularly polarized UV pulse, for example the 4th harmonic of a Ti:Sa laser, is sent to a sample of planar molecules such that its polarization, specified by the two orthogonal electric fields $\mathbf{E}_{\text{L1}}$ and $\mathbf{E}_{\text{L2}}$, is orthogonal to the static electric field. Hence the triple product $\mathbf{E}_{\text{static}} \cdot [\mathbf{E}_{\text{L1}} \times \mathbf{E}_{\text{L2}}] \neq 0$. The electric field tends to orient the molecules by their permanent dipole moment $\boldsymbol{\mu}_{00}$, while the UV pump pulse creates a coherent chiral vibrational wavepacket by resonant Raman excitation of the out-of-plane vibration. The resulting time-dependent chirality can be probed by a circularly polarized XUV probe pulse with the same propagation direction as the UV pump pulse. The probe ionizes the molecular ensemble, and photoelectron angular distributions (PAD) are measured using velocity map imaging (VMI). Recording PADs with both helicities of the probe pulse and taking their difference allows for quantifying the produced enantiomeric excess in terms of photoelectron circular dichroism (PECD). Figure S1 shows one possible configuration that would allow to use the static electric field of the VMI for $\mathbf{E}_{\text{static}}$. Other field configurations might exist that are more suitable for a given experimental setup.

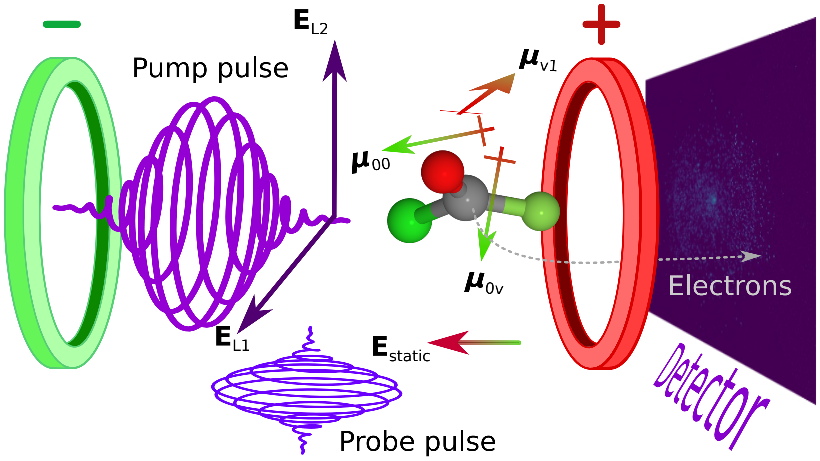

Figure S1 Example of a field configuration to realize the proposed pump-probe spectroscopy in an experiment. Electric field coils create the static electric field as well as the extraction field to image the photoelectrons resulting from the ionization of the planar molecules, taken to be COFCl here. The UV pump pulse prepares the vibrational superposition via a Raman excitation, whereas the probe pulse ionizes the (time-dependent) superposition.

B. Minimum number of fields to realize enantiomeric excess

To produce a broken parity, chiral state in a molecule, we need to create a superposition state from two (or more) levels of opposite parity [20]. One example of such states can be the vibrational states of the molecule, for instance the ground ($|0\rangle$) and first excited ($|1\rangle$) states of the out-of-plane (OOP) vibration of the COFCl molecule (for definition of the OOP coordinate see also Fig. S3). Let us assume the molecule to be initially in state $|0\rangle$. The superposition state after the excitation, for a single molecule with a particular orientation in space, is given by

$$|\psi(t)\rangle \approx |0\rangle + c_1^{(n)} \exp(-i\omega_{01}t)|1\rangle, \tag{1}$$

where $\hbar\omega_{01}$ the energy difference between the two levels. The coefficients $c_1^{(n)}$ are calculated with time-dependent perturbation theory of order n,

$$c_1^{(n)}(t) = -\frac{i}{\hbar}\sum_{v\neq 1}\int_{t''}^{t} dt' \langle 1|\hat{H}_{\text{int}}(t')|v\rangle \exp\left[-i\omega_{v1}(t'-t'')\right] c_v^{(n-1)}(t'). \tag{2}$$

Here, $\hbar\omega_{v1}$ is the energy difference between the vibrational states $|v \neq 1\rangle$ and $|1\rangle$. In particular, we consider direct excitation of the vibrational state $|1\rangle$ with an IR-pulse, which corresponds to first order perturbation theory ($n = 1$), and, alternatively, UV Raman excitation via virtual excited states, which is described by second order perturbation theory ($n = 2$). The laser-molecule interaction is given by

$$\hat{H}_{\text{int}}(t) = -\mathbf{E}(t)\, \mathcal{R}(\varphi,\theta,\chi)\, \hat{\boldsymbol{\mu}}\,, \tag{3}$$

where $\hat{\boldsymbol{\mu}}$ is the dipole moment in the molecular frame, $\mathbf{E}(t)$ the electric field in the laboratory frame, and $\mathcal{R}$ the rotation matrix connecting the two frames and depending on the Euler angles ϕ, θ, χ,

$$\mathcal{R}(\phi,\theta,\chi) = \underbrace{\begin{pmatrix}\cos(\chi) & -\sin(\chi) & 0\\ \sin(\chi) & \cos(\chi) & 0\\ 0 & 0 & 1\end{pmatrix}}_{\mathcal{R}_\chi} \cdot \underbrace{\begin{pmatrix}1 & 0 & 0\\ 0 & \cos(\theta) & -\sin(\theta)\\ 0 & \sin(\theta) & \cos(\theta)\end{pmatrix}}_{\mathcal{R}_\theta} \cdot \underbrace{\begin{pmatrix}\cos(\phi) & -\sin(\phi) & 0\\ \sin(\phi) & \cos(\phi) & 0\\ 0 & 0 & 1\end{pmatrix}}_{\mathcal{R}_\phi}. \tag{4}$$

For excitation with an IR pulse ($n = 1$) or a Raman pulse ($n = 2$), inserting Eq. (3) into Eq. (2) allows us to separate $c_1^{(n)}$ into

$$c_1^{(n)}(t) = \prod_{k=1}^{n}\left(\mathbf{E}_k \cdot \mathcal{R}\left(\varphi,\theta,\chi\right) \boldsymbol{\mu}_k\right) \tilde{c}_1^{(n)}(t)\,, \tag{5}$$

where $\tilde{c}_1^{(n)}(t)$ does not depend on the Euler angles and is calculated in detail in Sec. IC. The angular dependence is expressed by the product $\prod_{k=1}^{n}\left(\mathbf{E}_k \cdot \mathcal{R}\left(\varphi,\theta,\chi\right) \boldsymbol{\mu}_k\right)$, where $\mathbf{E}_k$ denotes the polarization direction and amplitude of the field responsible for the kth order interaction, and $\boldsymbol{\mu}_k = \langle v'|\hat{\boldsymbol{\mu}}|v\rangle$ is the corresponding transition matrix element. The time-dependent part $\tilde{c}_1^{(n)}(t)$ does not depend on the Euler angles and is calculated in detail in Sec. I.C. For simplicity, we assume here that in case of the UV Raman excitation the coefficients $c_1^{(n)}(t)$ result from a single intermediate state $|v\rangle$. A more general derivation of $c_1^{(n)}(t)$, including the sum over all intermediate states $|v\rangle$, is presented below in Sec. IC.

Observables for a molecule with given orientation are obtained as

$$\langle\mathcal{O}\rangle(t) = \langle\psi(t)|\hat{\mathcal{O}}|\psi(t)\rangle = \langle 0|\hat{\mathcal{O}}|0\rangle + |c_1^{(n)}|^2\langle 1|\hat{\mathcal{O}}|1\rangle + 2\langle 0|\hat{\mathcal{O}}|1\rangle \cdot \text{Re}\left[c_1^{(n)}\exp(-i\omega_{01}t)\right]\,. \tag{6}$$

We are interested in observables that are sensitive to the coherence of the state and assume without loss of generality $\langle 0|\hat{\mathcal{O}}|0\rangle = \langle 1|\hat{\mathcal{O}}|1\rangle = 0$ and $\langle 0|\hat{\mathcal{O}}|1\rangle \neq 0$. Then

$$\langle\mathcal{O}\rangle(t) = 2|c_1^{(n)}|\langle 0|\hat{\mathcal{O}}|1\rangle\cos(\omega_{01}t + \tilde{\varphi}_1) \tag{7}$$

with $c_1^{(n)} = |c_1^{(n)}| \cdot \exp(-i\tilde{\varphi}_1)$. In order to relate $\langle\mathcal{O}\rangle(t)$, i.e., the signal produced by a single molecule at a given orientation, to the macroscopic response of the system, we need to average over all the orientations that a molecule in the gaseous sample can take. We describe the rotational average classically,

$$\langle\langle\mathcal{O}\rangle\rangle(t) = \int d\Omega P(\phi,\theta,\chi)\langle\mathcal{O}\rangle(t)\,, \tag{8}$$

where $\int d\Omega = \int_0^{2\pi} d\phi \int_0^{\pi} \sin\theta d\theta \int_0^{2\pi} d\chi$ denotes the integration over the Euler angles. In the presence of a static electric field $\mathbf{E}_{\text{static}}$, the orientational distribution of the molecules in thermal equilibrium at temperature T is given by

$$P(\phi,\theta,\chi) = \frac{1}{Q}\exp\left(-\frac{E_{\text{rot}}}{k_{\text{B}}T}\right)\exp\left(\frac{\mathbf{E}_{\text{static}}\cdot\mathcal{R}(\phi,\theta,\chi)\boldsymbol{\mu}_{00}}{k_{\text{B}}T}\right), \tag{9}$$

where Q is the partition function, E_{rot} the (classical) rotational energy and $\boldsymbol{\mu}_{00}$ the permanent dipole moment of the molecule. Classical rotational averaging is valid as long as as the rotational spectrum is not resolved, i.e., for time-scales shorter than the rotational period. The rotational period of the molecules around the principle axis α ($\alpha = a, b, c$) can be estimated as $\tau_{\text{rot},\alpha} \propto B_\alpha^{-1}$, where B_α is the rotational constant for rotation about that principle axis. The shortest rotational period corresponds to the largest rotational constant, $B_{\text{a}} = A$. For COFCl, $A = 12\,\text{GHz}$, and thus $\tau_{\text{rot},a} = 8\,\text{ps}$. This is much longer than typical pump-probe delays of a few hundred femtoseconds. Moreover, we neglect a possible time-dependence of $P(\phi,\theta,\chi)$. This is justified if the interaction with the pump pulse is too weak to create a rotational wavepacket that causes pronounced alignment. To test this assumption, we have calculated the classical alignment factors of COFCl with a Monte-Carlo simulation for classical rigid rotors. We have found that a pump pulse with an intensity of $10^{13}\,\text{W/cm}^2$ and a FWHM of 150 fs indeed does not create molecular alignment within several ps, so that the assumption of a time-independent angular distribution function is justified.

Expanding the Boltzmann distribution to first order in the static field amplitude, the orientational distribution can be approximated by

$$P(\phi,\theta,\chi) \approx P^{(0)}\left(1 + P^{(1)}(\phi,\theta,\chi)\right), \tag{10}$$

where $P^{(0)} = \frac{1}{Q}\exp\left(-\frac{E_{\text{rot}}}{k_{\text{B}}T}\right)$ is the field-free isotropic distribution which does not depend on the Euler angles, while

$$P^{(1)}(\phi,\theta,\chi) = \frac{\mathbf{E}_{\text{static}}\cdot\mathcal{R}(\phi,\theta,\chi)\boldsymbol{\mu}_{00}}{k_{\text{B}}T} \tag{11}$$

describes the first-order orientation produced by the static field. Rotational Debye temperatures for COFCl, below which the thermal ensemble cannot be described classically anymore, are around 0.5 K. For rotational temperatures above 1 K, a classical treatment is thus well justified.

As a result, the orientational average for an observable $\mathcal{O}$ is given by

$$\langle\langle\mathcal{O}\rangle\rangle \propto 2|\tilde{c}_1^{(n)}(t)|\langle 0|\hat{\mathcal{O}}|1\rangle\cos(\omega_{01}t+\tilde{\varphi}_1)\int d\Omega\left(\prod_{k=1}^{n}(\mathbf{E}_k\cdot\mathcal{R}(\phi,\theta,\chi)\boldsymbol{\mu}_k)\right)\left[1+\frac{\mathbf{E}_{n+1}\cdot\mathcal{R}(\phi,\theta,\chi)\boldsymbol{\mu}_{n+1}}{k_{\text{B}}T}\right], \tag{12}$$

with $\mathbf{E}_{n+1} = \mathbf{E}_{\text{static}}$ and $\boldsymbol{\mu}_{n+1} = \boldsymbol{\mu}_{00}$. Evaluating the integrals according to Ref. [22], we find, depending on the number of external fields,

$N = 1$: $\int d\Omega(\mathbf{E}_1\cdot\mathcal{R}(\phi,\theta,\chi)\boldsymbol{\mu}_1) = 0$. This means that a single IR pulse (with $\mathbf{E}_1 = \mathbf{E}_{\text{L1}}$ and $\boldsymbol{\mu}_1 = \boldsymbol{\mu}_{01}$ in the notation of the main text), inducing the transition $|0\rangle \to |1\rangle$, cannot produce a chiral signature for the ensemble,

$N = 2$: $\int d\Omega\prod_{k=1}^{2}(\mathbf{E}_k\cdot\mathcal{R}(\phi,\theta,\chi)\boldsymbol{\mu}_k) \propto (\mathbf{E}_1\cdot\mathbf{E}_2)(\boldsymbol{\mu}_1\cdot\boldsymbol{\mu}_2)$. Such a configuration could be realized in two different ways with both resulting, however, in a vanishing integral:

- IR excitation $|0\rangle \to |1\rangle$ combined with static field orientation ($\mathbf{E}_1 == \mathbf{E}_{\text{L1}}$, $\mathbf{E}_2 = \mathbf{E}_{\text{static}}$, $\boldsymbol{\mu}_1 = \boldsymbol{\mu}_{01}$, and $\boldsymbol{\mu}_2 = \boldsymbol{\mu}_{00}$ in the notation of the main text). The integral vanishes because the permanent dipole moment $\boldsymbol{\mu}_{00}$ is in the molecular plane, whereas the transition dipole moment $\boldsymbol{\mu}_{01}$ is orthogonal to the molecular plane and therefore $(\boldsymbol{\mu}_{00}\cdot\boldsymbol{\mu}_{01}) = 0$.
- Raman excitation $|0\rangle \to |1\rangle$ without static field also does not yield a chiral signal ($\mathbf{E}_1 = \mathbf{E}_{\text{L1}}$, $\mathbf{E}_2 = \mathbf{E}_{\text{L2}}$, $\boldsymbol{\mu}_1 = \boldsymbol{\mu}_{0v}$, and $\boldsymbol{\mu}_2 = \boldsymbol{\mu}_{v1}$ in the notation of the main text). The integrand vanishes because the two transition dipole moments are necessarily orthogonal to each other, as explained in more detail below in Section I D.

$N = 3$: $\int d\Omega\prod_{k=1}^{3}(\mathbf{E}_k\cdot\mathcal{R}(\phi,\theta,\chi)\boldsymbol{\mu}_k) \propto \Big(\mathbf{E}_1\cdot[\mathbf{E}_2\times\mathbf{E}_3]\Big)\Big(\boldsymbol{\mu}_1\cdot[\boldsymbol{\mu}_2\times\boldsymbol{\mu}_3]\Big)$. These triple products do not necessarily vanish, which is why $N = 3$ is the smallest number of fields that may result in a non-zero rotational average. It requires three orthogonal (permanent or transition) dipole moments, with their triple product changing sign under spatial inversion. One possibility to realize the corresponding field configuration is Raman excitation with circular polarization in the presence of a static electric field perpendicular to the polarization plane of the circularly polarized field. $\mathbf{E}_1 = \mathbf{E}_{\text{L1}}$, $\mathbf{E}_2 = \mathbf{E}_{\text{L2}}$, $\mathbf{E}_3 = \mathbf{E}_{\text{static}}$, $\boldsymbol{\mu}_1 = \boldsymbol{\mu}_{0v}$, $\boldsymbol{\mu}_2 = \boldsymbol{\mu}_{v1}$, and $\boldsymbol{\mu}_3 = \boldsymbol{\mu}_{00}$ in the notation of the main text.

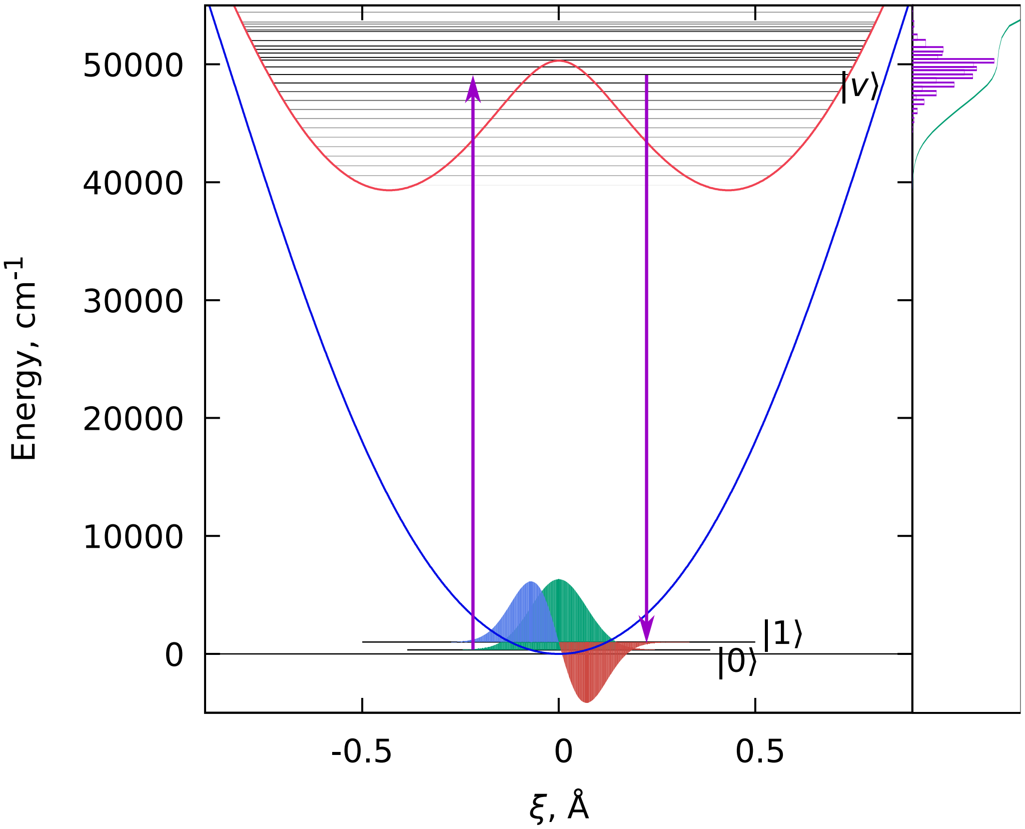

Figure S2 Raman excitation of vibrational states for COFCl: Ground and excited electronic potential surfaces along the out-of-plane coordinate ξ with vibrational levels (data shown for the example of the COFCl molecule). The purple arrows indicate the Raman excitation by the electric fields $\mathbf{E}_{\mathrm{L}1}$ and $\mathbf{E}_{\mathrm{L}2}$. The figure also shows the vibrational eigenfunctions $|0\rangle$ (green) and $|1\rangle$ (blue/red). The purple bars (green line) indicate the theoretical (experimental) UV absorption spectrum of COFCl, which is shown in more detail in Fig. S4.

C. Magnitude of the induced enantiomeric excess

After clarifying the conditions for observing enantiomeric excess in a sample of randomly oriented molecules, we now estimate its magnitude. To this end, we utilize time-dependent perturbation theory (TDPT). The electric field driving the Raman exciation is given by

$$\mathbf{E}(t) = E_{\mathrm{L}} \cdot (\mathbf{n}_1 \cos(\omega_{\mathrm{L}} t + \varphi_1) + \mathbf{n}_2 \cos(\omega_{\mathrm{L}} t + \varphi_2)) \cdot \exp\left(-\frac{t^2}{\tau^2}\right) \tag{13}$$

with $\mathbf{E}_{\mathrm{L}1} = E_{\mathrm{L}} \cdot \mathbf{n}_1$ and $\mathbf{E}_{\mathrm{L}2} = E_{\mathrm{L}} \cdot \mathbf{n}_2$. Here, E_{L} is the electric field peak amplitude, $\mathbf{n}$ and φ are the polarization axes and phases, ω_{L} is the pulse carrier frequency, and τ the pulse duration, related to the full width at half maximum (FWHM) by FWHM $= 2\sqrt{\ln(2)}\tau$. We consider Raman excitation from the ground to the first excited state of the out-of-plane vibration in the electronic ground state via vibronically excited states $|v\rangle$, $|0\rangle \to |v\rangle \to |1\rangle$, see Fig. S2. This requires TDPT to second order, with the corresponding wavefunction given by

$$|\psi\rangle(t) = |0\rangle + c_1^{(2)}(t)\exp(-i\omega_{01}t)|1\rangle + \sum_v c_v^{(1)}(t)\exp(-i\omega_{0v}t)|v\rangle\,,$$

where $\omega_{lm} = (E_m - E_l)/\hbar$ is the angular frequency of the $|l\rangle \to |m\rangle$ transition. Invoking the rotating wave approximation, the expansion coefficients become

$$\begin{aligned} c_m^{(n+1)}(t) &= -i \sum_{l\neq m} \frac{\mathcal{R}\overbrace{\langle m|\hat{\boldsymbol{\mu}}|l\rangle}^{\boldsymbol{\mu}_{lm}=\boldsymbol{\mu}_{ml}}}{\hbar} \int_{-\infty}^{t} \mathbf{E}(t')\cdot\exp(i\omega_{lm}t')\cdot c_l^{(n)}(t')dt' \qquad (14)\\ &\approx -i \sum_{l\neq m} \frac{\mathcal{R}\boldsymbol{\mu}_{lm}E_\mathrm{L}}{2\hbar}\big(\mathbf{n}_1\exp[-i\,\mathrm{sgn}(\omega_{lm})\varphi_1] + \mathbf{n}_2\exp[-i\,\mathrm{sgn}(\omega_{lm})\varphi_2]\big)\\ &\quad \cdot \int_{-\infty}^{t}\exp\left[-\frac{t'^2}{\tau^2} - i\Delta_{lm}t'\right]\cdot c_l^{(n)}(t')dt' \ , \end{aligned}$$

where $\Delta_{lm} = \omega_{lm} - \mathrm{sgn}(\omega_{lm})\cdot\omega_\mathrm{L}$ is the detuning and $\boldsymbol{\mu}_{lm}$ the transition dipole moment in the molecular frame, and $\mathcal{R} = \mathcal{R}(\phi,\theta,\chi)$ is the rotation matrix (Eq. (4)), transforming the molecular frame into the laboratory fame. Evaluation to first order yields the coefficients of the virtual states $|v\rangle$,

$$c_v^{(1)}(t) = -i\frac{E_\mathrm{L}}{2\hbar}\left\{\mathcal{R}\boldsymbol{\mu}_{0v}\left(\mathbf{n}_1\exp(-i\varphi_1)+\mathbf{n}_2\exp(-i\varphi_2)\right)\right\}\cdot\frac{\sqrt{\pi}\tau}{2}\left[1+\mathrm{erf}\left(\frac{t}{\tau}+i\frac{\tau\cdot\Delta_{0v}}{2}\right)\right]\cdot\exp\left(-\frac{\tau^2\Delta_{0v}^2}{4}\right) \ ,$$

where $\exp\left(-\frac{\tau^2\Delta_{0v}^2}{4}\right)$ represents the spectral profile of the Gaussian-shaped laser pulse. The second order coefficient cannot be evaluated analytically for non-vanishing detuning. We therefore estimate it approximately for zero detunings and account for the spectral pulse profile by multiplication with the two exponents of the form $\exp\left(-\frac{\tau^2\Delta^2}{4}\right)$ afterwards. This procedure is equivalent to treating the excitation as resonant absorption at the specific transition frequencies within the broad Gaussian spectrum of the pulse. With the time integral of the form

$$\int_{-\infty}^{x}\exp(-q^2)(1+\mathrm{erf}(q))dq = \frac{\sqrt{\pi}}{4}(1+\mathrm{erf}(x))^2 \ ,$$

this results in

$$\begin{aligned} c_1^{(2)}(t) \approx -\sum_v \frac{\pi\tau^2}{4}\left(1+\mathrm{erf}\left(\frac{t}{\tau}\right)\right)^2 \cdot \frac{E_\mathrm{L}^2}{4\hbar^2}\cdot\left\{\mathcal{R}\boldsymbol{\mu}_{0v}\left(\mathbf{n}_1\exp(-i\varphi_1)+\mathbf{n}_2\exp(-i\varphi_2)\right)\right\}\cdot\\ \cdot\left\{\mathcal{R}\boldsymbol{\mu}_{v1}\left(\mathbf{n}_1\exp(+i\varphi_1)+\mathbf{n}_2\exp(+i\varphi_2)\right)\right\}\cdot\exp\left(-\frac{\tau^2\Delta_{0v}^2}{4}-\frac{\tau^2\Delta_{1v}^2}{4}\right) \ . \qquad (15) \end{aligned}$$

Inserting Eq. (15) into Eq. (12) yields rotationally averaged expectation values in second order TDPT,

$$\begin{aligned} \langle\langle\mathcal{O}\rangle\rangle(t) &= \int_0^{2\pi}d\phi\int_0^{\pi}\sin(\theta)d\theta\int_0^{2\pi}d\chi\langle\mathcal{O}\rangle(t,\phi,\theta,\chi)P(\phi,\theta,\chi)\\ &= 2\langle 0|\hat{\mathcal{O}}|1\rangle\sin(\varphi_2-\varphi_1)\sin(\omega_{01}t)E_\mathrm{L}^2E_\mathrm{static}\frac{\pi\tau^2}{48\hbar^2k_\mathrm{B}T}\left(1+\mathrm{erf}\left(t/\tau\right)\right)^2\\ &\quad\cdot\sum_v\exp\left(-\left(\tau^2\Delta_{0v}^2+\tau^2\Delta_{1v}^2\right)/4\right)\left(\boldsymbol{\mu}_{00}\cdot[\boldsymbol{\mu}_{0v}\times\boldsymbol{\mu}_{1v}]\right)\left(\mathbf{n}_0\cdot[\mathbf{n}_1\times\mathbf{n}_2]\right) \ . \qquad (16) \end{aligned}$$

Equation (16) reveals how to maximize enantiomeric excess: The Raman pulse should be circularly polarized since $|\sin(\varphi_2-\varphi_1)| = 1$ is maximal for $|\varphi_2-\varphi_1| = \pi/2$, and the static electric field and polarization axes should all be mutually orthogonal to maximize $\mathbf{n}_0\cdot[\mathbf{n}_1\times\mathbf{n}_2] = 1$.

D. Symmetry considerations

In this Section, we employ symmetry arguments to determine which components of the dipole and transition dipole moments $\boldsymbol{\mu}_{00}$, $\boldsymbol{\mu}_{0v}$ and $\boldsymbol{\mu}_{v1}$ are non-zero. We consider planar molecules of C_s symmetry, with the ab-plane as symmetry plane and the molecular c-axis perpendicular to the symmetry-plane, then, the molecular axes $a, b \in A'$ and $c \in A''$.

The Cartesian components of the molecular dipole moments are the projections onto the molecular axes, $\mu^a \propto a$, $\mu^b \propto b$, $\mu^c \propto c$, and thus transform as the molecuar axis a, b and c. In order to determine the non-zero components of the dipole and transition dipole moments, we separate the OOP vibronic states $|0\rangle$, $|1\rangle$, and $|v\rangle$ into their vibrational and electronic part,

$$|0\rangle = |0\rangle_{\rm vib}|{\rm g}\rangle_{\rm el}\ ,\ \ |1\rangle = |1\rangle_{\rm vib}|{\rm g}\rangle_{\rm el}\ ,\ \ |v\rangle = |v\rangle_{\rm vib}|{\rm e}\rangle_{\rm el}\ .$$

The out-of-plane motion has A'' symmetry, which means that the symmetry of the vibrational states along the OOP coordinate is $\Gamma(|0\rangle_{\rm vib}) = A'$ and $\Gamma(|1\rangle_{\rm vib}) = A''$, i.e. the ground vibrational state is symmetric while the first excited state is anti-symmetric. Likewise, for the vibrational wavefunctions in the excited electronic state $\Gamma(|v\rangle_{\rm vib})$ is either A' or A'' for even v and odd v, respectively.

The components $i, j, k = a, b, c$ of the dipole and transition dipole moments are then

$$\begin{aligned}
\mu^i_{00} &= \langle 0|\hat{\mu}^i|0\rangle = \langle 0|_{\rm vib}\langle {\rm g}|_{\rm el}\hat{\mu}^i|{\rm g}\rangle_{\rm el}|0\rangle_{\rm vib}\ ,\\
\mu^j_{0v} &= \langle 0|\hat{\mu}^j|v\rangle = \langle 0|_{\rm vib}\langle {\rm g}|_{\rm el}\hat{\mu}^j|{\rm e}\rangle_{\rm el}|v\rangle_{\rm vib}\ ,\\
\mu^k_{v1} &= \langle v|\hat{\mu}^k|1\rangle = \langle v|_{\rm vib}\langle {\rm e}|_{\rm el}\hat{\mu}^k|{\rm g}\rangle_{\rm el}|1\rangle_{\rm vib}\ .
\end{aligned}$$

For each of the components to be nonzero, the product of the respective representations has to be fully symmetric, i.e. transform according to A' ($\Gamma_1 \otimes \Gamma_2 \otimes \ldots \otimes \Gamma_N = A'$). Since, for C_s symmetry, $A' \otimes A' = A'' \otimes A'' = A'$, and $A' \otimes A'' = A'' \otimes A' = A''$, this translates into

$$\begin{aligned}
\Gamma\left(\mu^i_{00}\right) &= \overbrace{\Gamma(\langle 0|_{\rm vib})}^{A'} \otimes \overbrace{\Gamma(\langle {\rm g}|_{\rm el})}^{A'} \otimes \Gamma(\hat{\mu}^i) \otimes \overbrace{\Gamma(|{\rm g}\rangle_{\rm el})}^{A'} \otimes \overbrace{\Gamma(|0\rangle_{\rm vib})}^{A'} = \Gamma(\hat{\mu}^i)\ ,\\
\Gamma\left(\mu^j_{0v}\right) &= \overbrace{\Gamma(\langle 0|_{\rm vib})}^{A'} \otimes \overbrace{\Gamma(\langle {\rm g}|_{\rm el})}^{A'} \otimes \Gamma(\hat{\mu}^j) \otimes \overbrace{\Gamma(|{\rm e}\rangle_{\rm el})}^{A''} \otimes \Gamma(|v\rangle_{\rm vib}) = \Gamma(\hat{\mu}^j) \otimes \Gamma(|v\rangle_{\rm vib}) \otimes A''\ ,\\
\Gamma\left(\mu^k_{v1}\right) &= \Gamma(\langle v|_{\rm vib}) \otimes \overbrace{\Gamma(\langle {\rm e}|_{\rm el})}^{A''} \otimes \Gamma(\hat{\mu}^k) \otimes \overbrace{\Gamma(|{\rm g}\rangle_{\rm el})}^{A'} \otimes \overbrace{\Gamma(|1\rangle_{\rm vib})}^{A''} = \Gamma(\hat{\mu}^k) \otimes \Gamma(|v\rangle_{\rm vib}) \otimes A'\ .
\end{aligned}$$

The excited electronic state is antisymmetric ($\Gamma(|{\rm e}\rangle_{\rm el}) = A''$) because the electronic transition is $n \to \pi^*$, and the π-orbitals are antisymmetric with respect to the plane of the molecule. The symmetry of dipole moment components is the same as for the molecular frame axes, i.e., $\mu^a, \mu^b \in A'$, whilst $\mu^c \in A''$. Thus, for the permanent dipole we have only μ^a_{00} and μ^b_{00} available for being nonzero, and $\mu^c_{00} = 0$. If the virtual state is symmetric $\Gamma(|v\rangle_{\rm vib}) = A'$, then the requirement is $\Gamma(\mu^j_{0v}) = \Gamma(\hat{\mu}^j) \otimes A''$ and $\Gamma(\mu^k_{v1}) = \Gamma(\hat{\mu}^k) \otimes A'$. These conditions can be fulfilled if $j = c$ and $k = a, b$. Similarly, if $\Gamma(|v\rangle_{\rm vib}) = A''$, then the conditions are inverted $\Gamma(\mu^j_{0v}) = \Gamma(\hat{\mu}^j) \otimes A'$ and $\Gamma(\mu^k_{v1}) = \Gamma(\hat{\mu}^k) \otimes A''$, which is fullfiled by $j = a, b$ and $k = c$.

Orientation-averaged enantiomeric excess requires the triple product

$$\boldsymbol{\mu}_{00} \cdot [\boldsymbol{\mu}_{0v} \times \boldsymbol{\mu}_{v1}] = \varepsilon_{ijk}\ \mu^i_{00}\ \mu^j_{0v}\ \mu^k_{v1}$$

to be nonzero where we have used Einstein notation with ε_{ijk} the Levi-Civita symbol, and $i, j, k = a, b, c$. Since $\varepsilon_{ijk} \neq 0$ only for $i \neq j \neq k \neq i$, the product is given by three different dipole moment components and these must be allowed to be non-zero by symmetry. Therefore, the following combinations are allowed by symmetry:

- if $\Gamma(|v\rangle_{\rm vib}) = A'$, then $\mu^a_{00}\mu^b_{0v}\mu^c_{v1}$ and $\mu^b_{00}\mu^a_{0v}\mu^c_{v1}$ can be nonzero,
- if $\Gamma(|v\rangle_{\rm vib}) = A''$, then $\mu^a_{00}\mu^c_{0v}\mu^b_{v1}$ and $\mu^b_{00}\mu^c_{0v}\mu^a_{v1}$ can be nonzero.

II. COMPUTATIONAL DETAILS FOR THE COFCL MOLECULE

A. Electronic structure

The quantum-chemical computations were performed at the B3LYP/cc-pVTZ level of theory [38, 39, 40, 42] using the Orca 4 package [44]. The structure of COFCl was optimized in the ground electronic state, yielding the equilibrium structure of the molecule given in Table S1 below. The harmonic frequency calculations confirmed the minimum. The ten lowest excited states were computed using time-dependent density functional theory within the Tamm-Dancoff approximation [43].

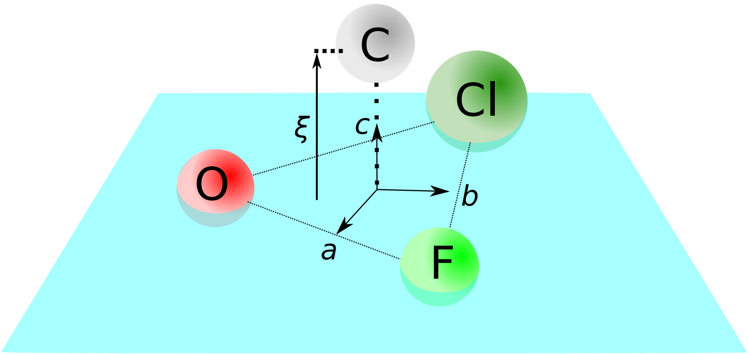

Figure S3 Definition of the out-of-plane coordinate: The molecular frame of the COFCl molecule is defined by the Cartesian coordinates a, b and c. The out-of-plane coordinate ξ is given by the distance of the C-atom from the molecular plane spanned by the O, F, and Cl atoms.

The molecular frame was chosen such that all atoms lie in the ab plane. The out-of-plane (OOP) coordinate ξ corresponds the c-coordinate of the carbon atom, while all the other atoms (O, F, and Cl) are assumed to be frozen at their equilibrium positions, cf. Figure S3. For the ground, first excited, and ground ionic potential energy surfaces (PES), 100 points were computed with $\xi \in [-0.9\text{Å}, +0.9\,\text{Å}]$. Along with the energies, the ground state dipole moment $\boldsymbol{\mu}_\mathrm{g}(\xi) = \langle \mathrm{g}(\xi)|_\mathrm{el}\hat{\boldsymbol{\mu}}|\mathrm{g}(\xi)\rangle_\mathrm{el}$ and transition dipole moment $\boldsymbol{\mu}_\mathrm{ge}(\xi) = \langle \mathrm{g}(\xi)|_\mathrm{el}\hat{\boldsymbol{\mu}}|\mathrm{e}(\xi)\rangle_\mathrm{el}$ were computed at each ξ. To provide the quantum-chemical results here in a compact form, the energies $V(\xi)$ and dipole moments $\mu(\xi)$ were approximated by polynomials

$$f(\xi) = \sum_{n=0}^{15} f_n \xi^n \,. \tag{17}$$

The approximation results are presented below in Section IV, with the polynomial coefficients listed in Tables S2, S3, and S4. The quality of the approximation can be judged from Figures S9, S10, and S11.

B. Vibrational structure

To calculate the vibrational states $|n\rangle_\mathrm{vib}$ of the OOP motion in the ground and first excited state, the grid points used in the electronic structure calculations were symmetrized along the mirror plane ($\xi = 0$) and then interpolated to a uniform grid of 1000 points using cubic splines. The reduced mass for the OOP motion is given by

$$\mu = \frac{m(^{12}\mathrm{C}) \cdot (m(^{16}\mathrm{O}) + m(^{19}\mathrm{F}) + m(^{35}\mathrm{Cl}))}{m(^{12}\mathrm{C}) + m(^{16}\mathrm{O}) + m(^{19}\mathrm{F}) + m(^{35}\mathrm{Cl})} \,.$$

With these parameters, the vibrational Schrödinger was solved for both electronic manifolds, using a discrete-variable representation [45]. The vibrational states in the electronically excited state $|v\rangle_\mathrm{vib}$ were the symmetrized with respect to $\xi = 0$. From the resulting vibronic wavefunctions, the vibrationally averaged permanent dipole moments $\boldsymbol{\mu}_{00} = \langle 0|_\mathrm{vib}\boldsymbol{\mu}_\mathrm{g}(\xi)|0\rangle_\mathrm{vib} = \langle 0|_\mathrm{vib}\langle \mathrm{g}|_\mathrm{el}|\hat{\boldsymbol{\mu}}|\mathrm{g}\rangle_\mathrm{el}|0\rangle_\mathrm{vib}$ and transition dipole moments $\boldsymbol{\mu}_{0v} = \langle 0|_\mathrm{vib}\boldsymbol{\mu}_\mathrm{ge}(\xi)|v\rangle_\mathrm{vib} = \langle 0|_\mathrm{vib}\langle \mathrm{g}|_\mathrm{el}|\hat{\boldsymbol{\mu}}|\mathrm{e}\rangle_\mathrm{el}|v\rangle_\mathrm{vib}$, $\boldsymbol{\mu}_{1v} = \langle 1|_\mathrm{vib}\boldsymbol{\mu}_\mathrm{ge}(\xi)|v\rangle_\mathrm{vib} = \langle 1|_\mathrm{vib}\langle \mathrm{g}|_\mathrm{el}|\hat{\boldsymbol{\mu}}|\mathrm{e}\rangle_\mathrm{el}|v\rangle_\mathrm{vib}$, corresponding to the Raman transition shown in Figure S2, were computed.

The quality of the vibronic states was evaluated by comparison with the available experimental data for $\mathrm{COF^{35}Cl}$. The experimental value for the out-of-plane fundamental vibrational transition ($|0\rangle \rightarrow |1\rangle$) is $667\,\mathrm{cm^{-1}}$, the value obtained in our calculations was $662\,\mathrm{cm^{-1}}$ (relative error of 1%). From the $|\boldsymbol{\mu}_{0v}|^2$ values, the UV absorption spectrum was also calculated. The comparison with the available band shape from the MPI-Mainz UV/VIS Spectral Atlas [50] shows a good reproduction of the first UV band, cf Fig. S4.

C. Vibrationally averaged photoelectron angular distributions

For the calculation of photoelectron angular distributions (PAD), we have extended the procedure of Refs. [29, 30] to explicitly account for the vibrational dynamics via vibrational averaging. Starting in the Born-Oppenheimer

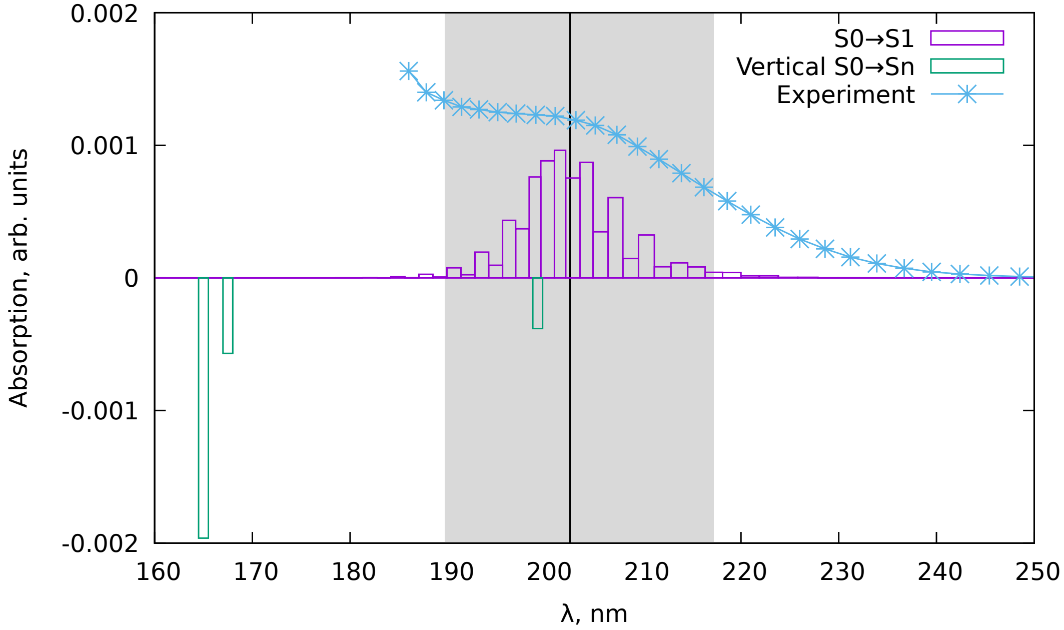

Figure S4 Absorption spectrum of COFCl: Experimental [50] (solid line with crosses) and calculated (violet boxes) vibronic absorption spectra of COFCl. The green boxes in the negative range illustrate positions and intensities of the vertical electronic spectrum computed from the equilibrium structure of the COFCl. The vertical line and the shaded area around it illustrate the position of the 4th harmonic of a Ti:Sa laser with a wavelength of 810 nm and the bandwidth of a Fourier-limited 10 fs pulse. The level of theory used was TDA-B3LYP/cc-pVTZ.

approximation, we write the vibronic state of the molecule as

$$|\psi\rangle(t) = \sum_j c_{j,\mathrm{g}}(t)\, e^{-i\omega_{j,\mathrm{g}}t}\ |j\rangle_{\mathrm{vib}}\, |\mathrm{g}\rangle_{\mathrm{el}} + \sum_v c_{v,\mathrm{e}}(t)\, e^{-i\omega_{v,\mathrm{e}}t}\ |v\rangle_{\mathrm{vib}}\, |\mathrm{e}\rangle_{\mathrm{el}} + \sum_{v'} c_{v',\mathbf{k}}(t)\, e^{-i\omega_{v',\mathbf{k}}t}\ |v'\rangle_{\mathrm{vib}}\, |\mathbf{k}\rangle_{\mathrm{el}}\ , \tag{18}$$

where $|\mathrm{g}\rangle_{\mathrm{el}}$ refers to the electronic ground state, $|\mathrm{e}\rangle_{\mathrm{el}}$ to the electronically excited state, and $|\mathbf{k}\rangle_{\mathrm{el}}$ is the electronic state of the ion after photoionization with $\mathbf{k} = (k, \phi_k, \theta_k)$ the laboratory frame momentum of the emitted electron. $|v\rangle_{\mathrm{vib}}$ represent the vibrational eigenstates in the corresponding PES with eigenfrequencies $\omega_{v,n}$. The expansion coefficients of the electronic ground state, $c_{j,\mathrm{g}}(t) = \sum_n c_{j,\mathrm{g}}^{(n)}(t)$ (with $c_{j,\mathrm{g}}^{(n)}(t)$ introduced in Sec. I C), describe the chiral vibrational wavepacket. We evaluate them up to second-order time-dependent perturbation theory according to Equation (14), describing the Raman excitation by the pump pulse. Here, we solve the second-order time integral numerically. Assuming one-photon ionization from the highest occupied molecular orbital by the circularly polarized XUV probe pulse $\mathbf{E}_{\mathrm{I}}$, we calculate the ionic expansion coefficients in Equation (18) by third-order perturbation theory,

$$c_{v',\mathbf{k}}^{(3)}(t) = -i \sum_{j=0,1} \int_{-\infty}^{t} e^{i(\omega_{v',\mathbf{k}}-\omega_{j,\mathrm{g}})t'}\ \langle v'|_{\mathrm{vib}}\, \langle \mathbf{k}|_{\mathrm{el}}\, \mathbf{E}_{\mathrm{I}}(t') \cdot \mathcal{R}(\phi,\theta,\chi) \cdot \hat{\boldsymbol{\mu}}\, |\mathrm{g}\rangle_{\mathrm{el}}\, |j\rangle_{\mathrm{vib}}\ c_{j,\mathrm{g}}^{(2)}(t')\, \mathrm{d}t'\ , \tag{19}$$

where $\mathcal{R}(\phi,\theta,\chi)$ transforms the molecule-fixed dipole operator into the laboratory-fixed frame of reference. Hence, the expansion coefficients depend on the orientation of the molecule, i.e., $c_{v,n}(t) \equiv c_{v,n}(t;\phi,\theta,\chi)$ for $n = \mathrm{g}, \mathrm{e}, \mathbf{k}$.

Equation (19) implies an integral over the vibrational coordinate,

$$\langle v'|_{\mathrm{vib}}\, \langle \mathbf{k}|_{\mathrm{el}}\, \hat{\boldsymbol{\mu}}\, |\mathrm{g}\rangle_{\mathrm{el}}\, |j\rangle_{\mathrm{vib}} = \int \chi_{v',\mathbf{k}}^{*}(\xi)\, \boldsymbol{\mu}_{\mathbf{k}\mathrm{g}}(\xi)\, \chi_{j,\mathrm{g}}(\xi)\, \mathrm{d}\xi\ ,$$

where $\chi_{j,\mathrm{g}}(\xi)$ and $\chi_{v',\mathbf{k}}(\xi)$ are the vibrational eigenfunctions in the neutral and ionic ground electronic states. In other words, the vibrational dynamics is taken into account by averaging over the parametric dependence of the electronic states on the nuclear coordinates, with the approximation that all nuclei are fixed except for the OOP motion, see Fig. S3. For a given, fixed value of the OOP coordinate ξ, we follow the procedure of Refs. [29, 30] to evaluate the electronic dipole matrix elements in Equation (19), $\boldsymbol{\mu}_{\mathbf{k}\mathrm{g}}(\xi) = \langle \mathbf{k}(\xi)|_{\mathrm{el}}\, \hat{\boldsymbol{\mu}}\, |\mathrm{g}(\xi)\rangle_{\mathrm{el}}$, by applying a single center expansion. This yields the transition dipole moments to the electronic continuum state of the ion in the frozen-core Hartree-Fock

approximation. The calculation is performed with the `ePolyScat` program. It requires the electronic ground state to be represented by a single Slater determinant [54, 55], which we calculate at the HF/aug-cc-pVTZ level of theory. Thus, the continuum dipole matrix elements are calculated for different values of ξ by manipulating the molecular geometry in the calculation of the ground state Slater determinant. This enables us to obtain vibronic transition dipole matrix elements in terms of the overlap of vibrational nuclear wavefunctions, calculated as described in Sec. II B, and the parametrized electronic dipole matrix elements. We use partial waves up to $L = 80$ to represent the bound orbitals on the angular grid in order to ensure converged transition dipole matrix elements at photoelectron energies up to 20 eV.

In order to reduce the numerical effort in evaluating Equation (19), we approximate the shape of the ionizing pulse by $\delta(t - t_I)$ and assume the PAD to display a Gaussian energy dependence, which is given by the ionizing pulse spectrum [56]. This neglects the energy dependence of the continuum dipole matrix elements. We have verified that this is a reasonable approximation as long as the probe pulse duration is significantly shorter than the oscillation period of enantiomeric excess, i.e., the ionization pulse is not longer than 5 fs. Of course, the latter is required in any case, in order to resolve the chiral vibrational motion.

For a single molecule with given orientation, the laboratory-frame PAD at time t for a photoelectron with momentum $\mathbf{k}$ is given in terms of the ionic expansion coefficients,

$$\mathrm{PAD}(t;\mathbf{k};\phi,\theta,\chi) = \sum_{v'} \left| c^{(3)}_{v',\mathbf{k}}(t;\phi,\theta,\chi)\right|^2 , \tag{20}$$

where the sum runs over all populated vibrational states v' in the ground electronic state of the ion. The orientation-averaged laboratory-frame PAD is then obtained by integrating over the Euler angles,

$$\langle \mathrm{PAD}\rangle(t;\mathbf{k}) = \int_0^{2\pi} d\phi \int_0^{\pi} \sin\theta \, d\theta \int_0^{2\pi} d\chi \, P(\phi,\theta,\chi)\,\mathrm{PAD}(t;\mathbf{k};\phi,\theta,\chi) , \tag{21}$$

where $P(\phi,\theta,\chi)$ describes the orientational distribution of the molecules, including the effect of the static electric field, cf. Sec. I B. As explained in Sec. I B, it is well justified to take the orientational distribution to be time-independent — this applies also to duration of the probe pulse. The integral over molecular orientations in Equation (21) is evaluated numerically.

Photoelectron circular dichroism (PECD) is the forward-backward asymmetry visible in the dichroic difference of PADs upon ionization with left- and right-circularly polarized (LCP/RCP) light. We quantify PECD according to the convention of Ref. [24], i.e.

$$\langle \mathrm{PECD}\rangle(t;\mathbf{k}) = \frac{\langle \mathrm{PAD}^{\mathrm{LCP}}\rangle(t;\mathbf{k}) - \langle \mathrm{PAD}^{\mathrm{RCP}}\rangle(t;\mathbf{k})}{(\langle \mathrm{PAD}^{\mathrm{LCP}}\rangle(t;\mathbf{k}) + \langle \mathrm{PAD}^{\mathrm{RCP}}\rangle(t;\mathbf{k}))/2} . \tag{22}$$

This convention is commonly used in experiments and often combined with an integration of the PADs over the forward and backward hemisphere [12, 25, 26, 27]. The latter can result in smaller values, if the individual hemispheres contain contributions to PECD with opposite signs, which cancel each other upon integration. In the proposed experiment, we observe almost complete cancellation of PECD inside the hemispheres with the pulse parameters given in the main text. In our understanding, this cancellation happens by chance for COFCl at the selected photoelectron energy, since there is no symmetry requirement that forces this cancellation to occur.

The orientation-averaged PADs are cylindrically symmetric due to the cylindrical symmetry of the proposed experimental setup (cf. Fig. S1). Hence, it does not matter if PECD is calculated from the PADs itself or from velocity map images (VMI), i.e. the projection of Eq. (22) onto the yz-plane. However, due to the orientation of the molecules by the static electric field, the dichroic differences are not antisymmetric with respect to the light propagation direction as it is the case for randomly oriented molecules. For completeness, Fig. S5 shows polar plots of the orientation-averaged laboratory frame PADs for the pump-probe delays that are marked in Fig. 3 of the main text, displaying PADs obtained with a left- and a right-circularly polarized probe pulse and their normalized dichroic difference.

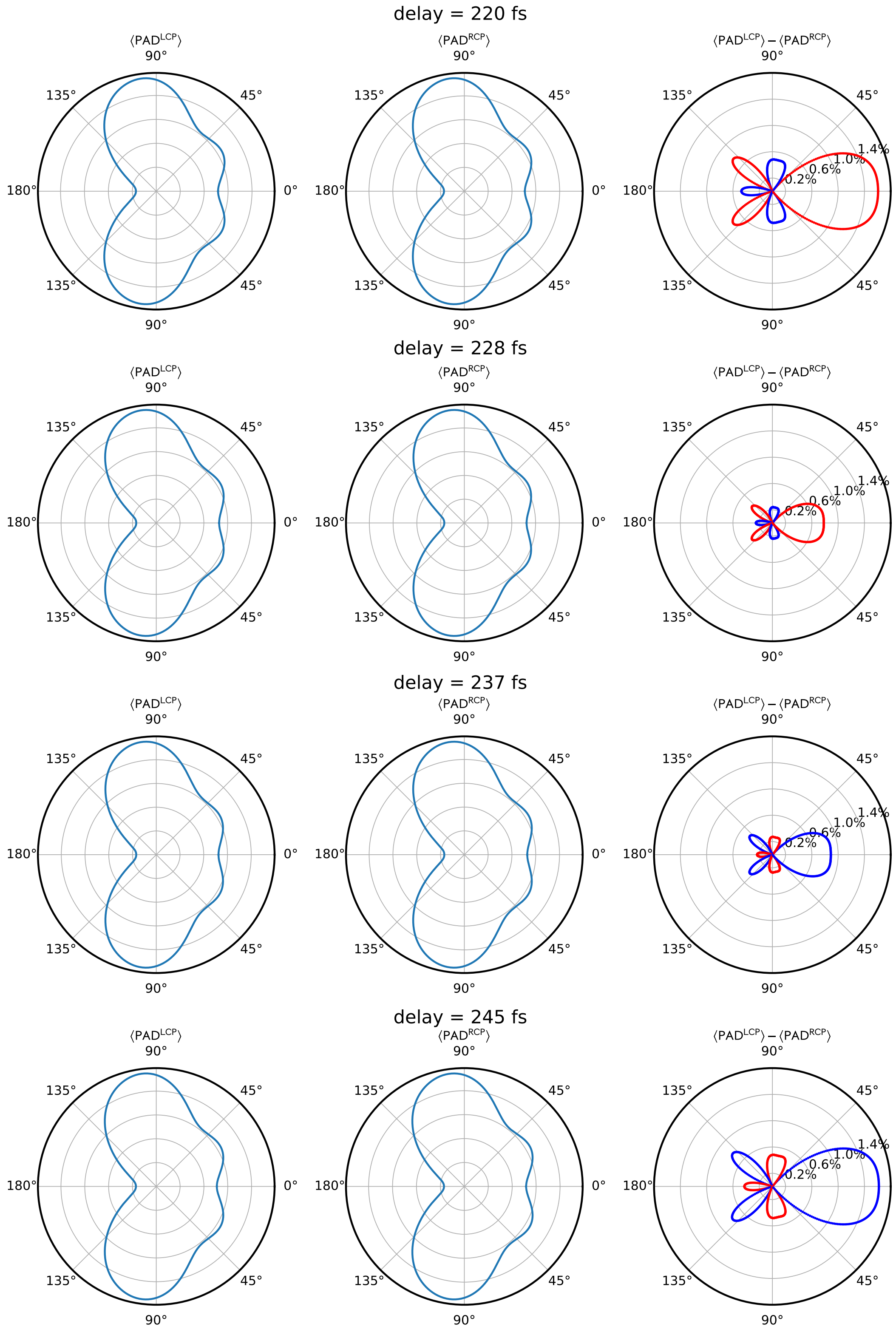

Figure S5 Photoelectron angular distribution: Polar plots of the orientation-averaged laboratory frame photoelectron angular distributions obtained with a left- and right-circularly polarized ionizing pulse and the corresponding dichroic difference where θ_k is the polar angle of the photoelectron momentum. The latter has been normalized with respect to the mean value of the corresponding PADs according to Eq. (22). The simulation parameters are the same as for the results presented in Fig. 3 of the main text. The rows correspond to the time delays that are marked with dashed vertical lines in Fig. 3 of the main text. The dichroic difference in the right column is shown as absolute value in percent, while the sign is encoded in the color, i.e., red and blue corresponds to positive and negative values, respectively.

III. DEPHASING AND RELAXATION TIMESCALES

An essential aspect of the ultrafast experiments is the decay time of the signal, because it limits the timescale over which the probe can monitor the effect induced by the pump pulse. Here, we consider mechanisms that may limit observation of the vibrational coherence. To observe the motion of the chiral wavepacket, at least one period of the out-of-plane vibration in COFCl, which is about 50 fs. Ideally, the shortest dephasing time should be at least an order of magnitude larger. Since we also assume pump and probe not to overlap in time, all the dephasing times should be larger than 1 ps in order to exclude detrimental effects of dephasing in the proposed experiment.

A. Collisional dephasing

Ultrafast experiments typically use either an effusive beam or supersonic expansion, both of which are hard to model. Instead, one can estimate an upper bound to the collision frequency by using a simple equilibrium gas model at elevated temperatures.

The collisional frequency in the ideal gas at equilibrium is given by [59]

$$\frac{1}{\tau_{\rm coll}} = 4\sigma \frac{P}{\sqrt{\pi m k_{\rm B} T}} \ ,$$

where $\tau_{\rm coll}$ is the collision period, σ the collisional cross-section, m the mass of the molecule, P pressure, T temperature, and $k_{\rm B}$ Boltzmann's constant. The upper boundary for the $\tau_{\rm coll}$ can be estimated by taking the $P = 1$ atm., $T = 300$ K, and $\sigma = \pi R^2$, where $R = (\overbrace{R(\mathrm{Cl}\ldots\mathrm{O})}^{2.6} + \overbrace{R_{\rm VdW}(\mathrm{Cl})}^{1.8} + \overbrace{R_{\rm VdW}(\mathrm{O})}^{1.5})/2 \approx 3$ Å with $R(\mathrm{Cl}\ldots\mathrm{O})$ being the distance between Cl and O in COFCl (the largest interatomic distance), and $R_{\rm VdW}$ are the van der Waals radii of the respective atoms [60]. Such estimation gives $\tau_{\rm coll} \approx 400$ ps, which is much greater than the desired limit of 1 ps.

B. Rotational dephasing

To estimate the timescale of rotational dephasing, we assume rotations and vibrations to be decoupled due to their different characteristic timescales and all vibrational-rotational transitions having the same transition matrix elements. The oscillation of the chiral observable $\mathcal{O}$ (Eq. 16) at long times after the pump pulse ($t \gg \tau$, when $(1+\mathrm{erf}(t/\tau)) \approx 2$) can be expressed as

$$I(t) = A \cdot \sin(\omega_{01} t) \ ,$$

where I is the intensity of the signal, ω_{01} the vibrational transition frequency in the electronic ground state, and A amplitude of the observable's ($\mathcal{O}$) oscillation. However, the vibrational coherence, induced by the laser pulse would include multiple rotational states. In the rigid rotor and harmonic oscillator (RRHO) approximation, the induced rovibrational transition frequency will be given as $\omega = \omega_{01} + \delta\omega$, where $\delta\omega$ denotes the difference in the rotational frequencies of the pair of the coherent states. We approximate the rotational states of the asymmetric top by those of a spherical top: Each state $|JKM\rangle$ with total momentum $J = 0, 1, 2, \ldots$, and projections of the total momentum on the molecular and lab frames $K, M = -J, -J+1, \ldots, 0, \ldots, J-1, J$ has an energy of $hBJ(J+1)$, where $B = \frac{\hbar}{4\pi I}$ is the rotational constant, dependent on the moment of inertia I. The rotational energy levels for each J are $(2J+1)^2$ times degenerate. We take the levels to be occupied according to Boltzmann distribution and assume that the classical regime is applicable, i.e., thermal fluctuations $k_{\rm B}T$ are more prominent than the difference between nearest quantum levels. With these assumptions, the predicted signal for the molecule in the specific initial and final rotational states will be given by

$$I = A \cdot \sin(\underbrace{\omega_{01} t}_{X = X(t)} + \delta\omega t) = A \cdot \sin(X + \delta\omega t) \ .$$

Vibrational-rotational spectra of nonlinear molecules in the electric dipole approximation consist of three branches of transitions with different frequency shifts due to rotational state change:

- P-branch with $\Delta J = -1$ ($|J\rangle \to |J-1\rangle$): $\delta\omega_P = -4\pi BJ$,
- Q-branch with $\Delta J = 0$ ($|J\rangle \to |J\rangle$): $\delta\omega_Q = 0$,

- R-branch with $\Delta J = +1$ ($|J\rangle \to |J+1\rangle$): $\delta\omega_R = 4\pi B(J+1)$.

For the Raman transition, the P- and R-branches have to be replaced by the O-branch ($|J\rangle \to |J-2\rangle$) and S-branch ($|J\rangle \to |J+2\rangle$) but in the classical limit ($\Delta E_{J\to J'} \approx J$) this will not change the final result.

The final signal will be a combination of interfering signals from each of the three branches,

$$I = \frac{1}{3}\left(\underbrace{\frac{A}{Z}\sum_{J=0}^{+\infty} w_J \sin(X - 4\pi BJt)}_{I_P} + \underbrace{\frac{A}{Z}\sin(X)\overbrace{\sum_{J=0}^{+\infty} w_J}^{Z}}_{I_Q} + \underbrace{\frac{A}{Z}\sum_{J=0}^{+\infty} w_J \sin(X + 4\pi B(J+1)t)}_{I_R}\right) = \frac{1}{3}\left(I_P + I_R + \underbrace{A\cdot\sin(X)}_{I_Q}\right) .$$

In this approximation, the signal from the Q-branch does not depend on J, whilst the frequencies of the P- and R-branches depend linearly on J. To estimate $I_P + I_R$, we take the classical limit, replacing the sum $\sum_{J=0}^{+\infty}$ by an integral $\int_0^{+\infty} dJ$ and assuming J to be large enough such that $J(J+1) \approx J^2$, $(2J+1)^2 \approx 4J^2$ and $J+1 \approx J$. This gives

$$\begin{aligned} I_P + I_R &\approx \frac{4A}{Z}\int_{J=0}^{+\infty} J^2 \exp\left(-\frac{hB}{k_\mathrm{B}T}J^2\right)\sin(X - 4\pi BJt)dJ + \frac{4A}{Z}\int_0^{+\infty} J^2 \exp\left(-\frac{hB}{k_\mathrm{B}T}J^2\right)\sin(X + 4\pi BJt)dJ \\ &= \frac{4A}{Z}\int_{-\infty}^{+\infty} J^2 \exp\left(-\frac{hB}{k_\mathrm{B}T}J^2\right)\sin(X + 4\pi BJt)dJ = \frac{4A}{Z}\frac{(I_+ - I_-)}{2i} \end{aligned}$$

with

$$I_+ = \int_{-\infty}^{+\infty} J^2 \exp\left(-\frac{hB}{k_\mathrm{B}T}J^2 + iX + i4\pi BJt\right)dJ\,, \quad I_- = \int_{-\infty}^{+\infty} J^2 \exp\left(-\frac{hB}{k_\mathrm{B}T}J^2 - iX - i4\pi BJt\right)dJ\,.$$

All the three integrals needed (Z, $I_\pm$) are easy to calculate:

$$\begin{aligned} Z &\approx 4\int_{J=0}^{+\infty} J^2 \exp\left(-\frac{hB}{k_\mathrm{B}T}J^2\right)dJ = \sqrt{\pi}\cdot\left(\frac{k_\mathrm{B}T}{hB}\right)^{3/2} . \\ I_\pm &= \exp(\pm iX)\cdot Z\cdot\exp\left(-\frac{2\pi k_\mathrm{B}TBt^2}{\hbar}\right)\cdot\left(\frac{1}{2} - (2\pi Bt)^2\right) , \end{aligned}$$

where for $I_\pm$ we have used the substitution $J \to j_\pm = J \mp \frac{ik_\mathrm{B}Tt}{\hbar}$. When combining all the terms, we obtain

$$I(t) = \frac{1}{3}\left(I_P(t) + I_R(t) + I_Q(t)\right) = A\cdot\sin(\underbrace{\omega_0 t}_{X})\cdot\underbrace{\frac{1}{3}\left(1 + 4\exp\left(-\frac{2\pi k_\mathrm{B}TBt^2}{\hbar}\right)\cdot\left(\frac{1}{2} - (2\pi Bt)^2\right)\right)}_{\Xi(t)} . \tag{23}$$

The maximal value of $\Xi(t)$ is one (for $t = 0$)), and the $t = +\infty$ limit is $\Xi(+\infty) = 1/3$, which corresponds to a cancelation of the signals between P and R branches with only the Q branch left. A plot of $\Xi(t)$ for the different rotational As an estimate of the rotational decoherence times, we can use the time of the half-decay $\tau_{\mathrm{rot},1/2}$ for the $\Xi(t)$, defined through the equation $\Xi(\tau_{\mathrm{rot},1/2}) = 2/3$.

The Figure S7 shows the dependence of the $\tau_{\mathrm{rot},1/2}$ for $COF^{35}Cl$ molecule at the different rotational temperatures. A signal lasting longer than 1 ps is expected for rotational temperatures below 70 K. Such temperatures are easily achieved in molecular beam experiments. In the proposed experiment, they are needed also to ensure negligible thermal population in the excited vibrational level of the electronic ground state. We therefore conclude that rotational dephasing will not be relevant.

C. Vibrational excitation intramolecular vibrational energy redistribution lifetime

Coupling of the degree of interest, the OOP vibration, to other vibrational modes will lead to intramolecular vibrational energy redistribution (IVR) and decay of the desired signal. To estimate the rate with which excitation in the OOP vibration decays into other vibrations due to anharmonic couplings, we have performed *ab initio* molecular dynamics (AIMD) simulations for COFCl. We have taken the $\langle 0|\xi|1\rangle$ average structure obtained at the B3LYP/cc-pVTZ level of

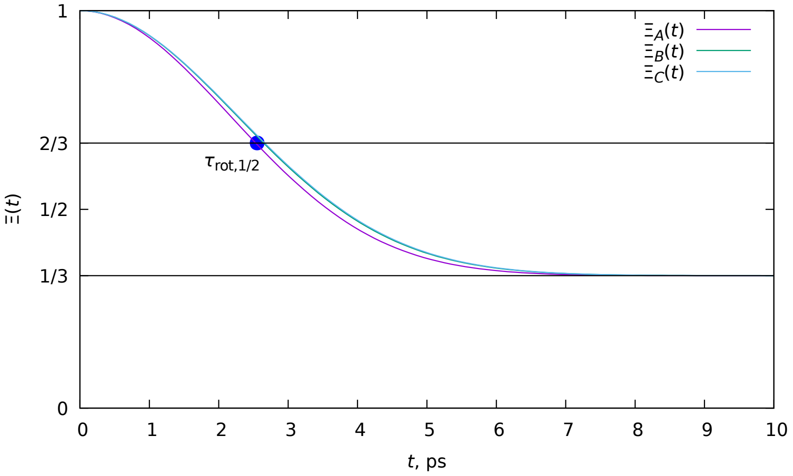

Figure S6 Rotational dephasing: Time-dependence of the rotational dephasing function $\Xi(t)$ (Equation (23)) at rotational temperature of 10 K for three rotational constants of $COF^{35}Cl$ molecule ($A = 11830\,\text{MHz}$, $B = 5287\,\text{MHz}$, and $C = 3649\,\text{MHz}$ taken from Ref. [49]). The point $\tau_{\text{rot},1/2}$ indicates the half-decay time for this $\Xi(t)$.

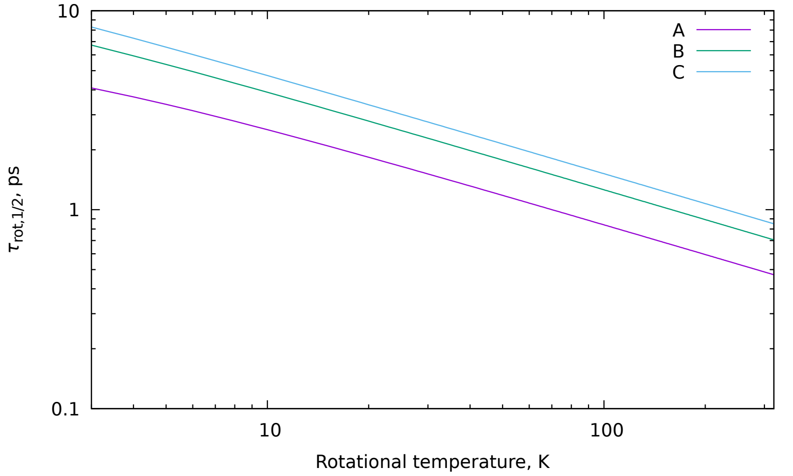

Figure S7 Temperature dependence of the rotational dephasing: The dependence of the half-decay times for the rotational dephasing function $\Xi(t)$ (Equation (23)), as a function of rotational temperature for the $COF^{35}Cl$ molecule with rotational constants $A = 11830\,\text{MHz}$, $B = 5287\,\text{MHz}$, and $C = 3649\,\text{MHz}$ (values taken from Ref. [49]).

theory, cf. Table S1, as the initial structure for the simulations. Then, 28 AIMD trajectories with velocities generated from a Maxwell-Boltzmann distribution at 300 K were calculated for a duration of 0.5 ps with a time step of 0.1 fs, such that the trajectories covered approximately ten periods of OOP vibration in COFCl. The gradients were obtained at the B3LYP-D3/def2-SVP level of theory. From each trajectory, the evolution of the carbon displacement from the ab-plane,

$$\xi = \frac{\mathbf{r}_{\text{OC}} \cdot [\mathbf{r}_{\text{OF}} \times \mathbf{r}_{\text{OCl}}]}{||[\mathbf{r}_{\text{OF}} \times \mathbf{r}_{\text{OCl}}]||}, \tag{24}$$

was extracted. The results are shown in Figure S8. The average time dependence of this coordinate was fitted to a function describing an harmonic oscillator with friction,

$$\xi(t) = A_\xi \cdot \sin(\omega_{01} t + \phi) \cdot \exp(-t/\tau_{\text{IVR}}) \tag{25}$$

with A_ξ the vibrational amplitude, ϕ the phase and τ_{IVR} an estimate of the IVR relaxation time, also shown in Figure S8. The latter was found to be $\tau_{\text{IVR}} = 3.6 \pm 0.2\,\text{ps}$. This suggests that IVR in COFCl is about two orders of

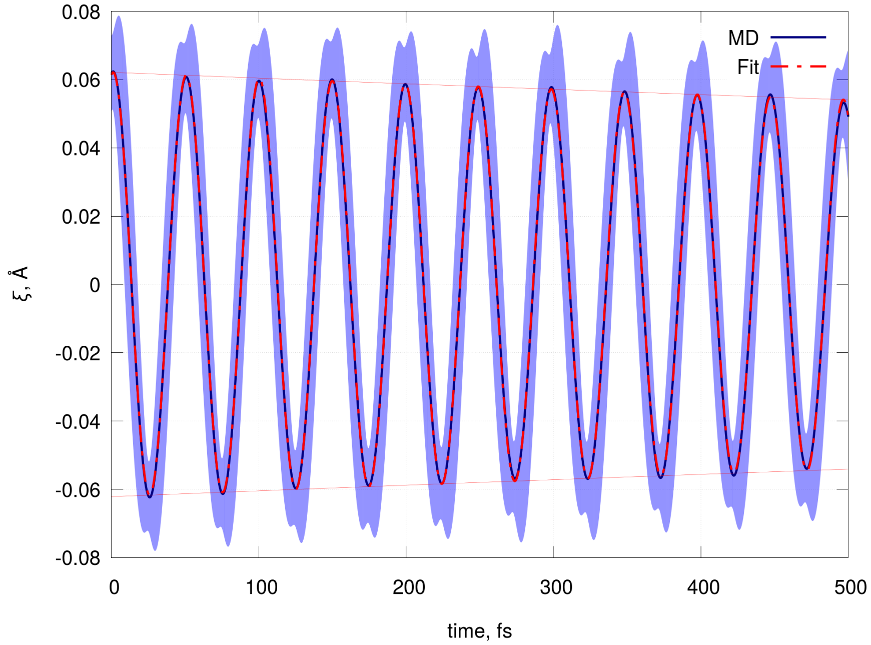

Figure S8 Decay of the vibrational amplitude due to vibrational energy redistribution: Average displacement of the ξ out-of-plane coordinate in COFCl (solid blue line) obtained with AIMD trajectory simulations at the B3LYP-D3/def2-SVP level of theory, cf. Equation (24). The average is fitted to Equation (25) (thick red line) which resuls in an estimated IVR time of $\tau_{\mathrm{IVR}} = 3.6 \pm 0.2\,\mathrm{ps}$. The light-blue area corresponds to the standard deviation of the ensemble of trajectories. The narrow red lines show the exponential decay of the vibrational amplitude.

magnitude larger than the period of the desired chiral vibrational dynamics and should thus not obstruct observation of the chiral signal.

IV. NUMERICAL DETAILS OF QUANTUM-CHEMICAL CALCULATIONS

Table S1 Equilibrium geometry of the COFCl at the B3LYP/cc-pVTZ level of theory and the geometry of the turning point for the vibrational wavepacket ($\langle 0|\xi|1\rangle$). The geometries are reported in XYZ file format with coordinates are given in Ångström.

```
4
 Equilibrium structure at B3LYP/cc-pVTZ level
Cl      1.2424559624     0.0064155674     0.0000000000
C      -0.4970547972     0.1473235259     0.0000000000
F      -1.0346338274    -1.0679815724     0.0000000000
O      -1.1144924363     1.1439709190     0.0000000000

4
 <0|xi|1> turning point structure
Cl      1.2424559624     0.0064155674     0.0000000000
C      -0.4970547972     0.1473235259     0.0498566330
F      -1.0346338274    -1.0679815724     0.0000000000
O      -1.1144924363     1.1439709190     0.0000000000
```

Table S2 The ground and excited potential energy surfaces of the neutral COFCl (V_{g} and V_{e}) and ground state potential of the COFCl cation (V_{i}) along the out-of-plane vibrational coordinate ξ (see also figure S9) at the B3LYP/cc-pVTZ and TDA-B3LYP/cc-pVTZ levels of theory. The approximation is made using polynomial given in Equation 17. Coefficients V_n are given in cm^{-1}.

n	$V_{\mathrm{g},n}$	$V_{\mathrm{e},n}$	$V_{\mathrm{i},n}$
0	-5.225	50203.464	98399.653
1	0.000	0.000	0.000
2	65964.258	-217734.795	16016.701
3	0.000	0.000	0.000
4	37689.075	2212090.458	2872.946
5	0.000	0.000	0.000
6	-27411.772	-14788725.875	2005.847
7	0.000	0.000	0.000
8	-256582.066	62737810.703	-9781.540
9	0.000	0.000	0.000
10	894591.518	-154205083.960	17659.820
11	0.000	0.000	0.000
12	-1299431.830	200002541.361	-19097.394
13	0.000	0.000	0.000
14	726023.329	-105709357.157	9364.166
15	0.000	0.000	0.000

Table S3 The ground state dipole moment dependence of the COFCl along the out-of-plane vibrational coordinate ξ at the B3LYP/cc-pVTZ level of theory. The approximation is made using polynomial given in Equation 17. Coefficients $\mu^i_{\mathrm{g},n},\ i = a, b, c$ are given in debyes (D).

n	$\mu^a_{\mathrm{g},n}$	$\mu^b_{\mathrm{g},n}$	$\mu^c_{\mathrm{g},n}$
0	-0.9314524903	-0.7624595494	0.0000000000
1	0.0000000000	0.0000000000	1.9073073452
2	-0.5087896375	1.3252089900	0.0000000000
3	0.0000000000	0.0000000000	-1.0960792347
4	0.8538036739	0.3899422020	0.0000000000
5	0.0000000000	0.0000000000	0.0820464672
6	0.3526400471	-0.4585282449	0.0000000000
7	0.0000000000	0.0000000000	0.8162906684
8	-1.6358233159	1.2532774805	0.0000000000
9	0.0000000000	0.0000000000	-0.6877409655
10	2.3283039851	-2.5396816022	0.0000000000
11	0.0000000000	0.0000000000	-0.2611082534
12	-2.2711754646	2.2187633865	0.0000000000
13	0.0000000000	0.0000000000	0.6187223861
14	0.7706536394	-0.7867363304	0.0000000000
15	0.0000000000	0.0000000000	-0.2737050963

Table S4 The ground to the first excited state transition dipole moment dependence of the COFCl along the out-of-plane vibrational coordinate ξ at the TDA-B3LYP/cc-pVTZ level of theory. The approximation is made using polynomial given in Equation 17. Coefficients $\mu^i_{\mathrm{ge},n},\ i = a, b, c$ are given in debyes (D).

n	$\mu^a_{\mathrm{ge},n}$	$\mu^b_{\mathrm{ge},n}$	$\mu^c_{\mathrm{ge},n}$
0	0.0000000000	0.0000000000	0.1326949748
1	-3.3639023583	-0.5049714658	0.0000000000
2	0.0000000000	0.0000000000	-1.0422105702
3	40.7810786314	2.4622616344	0.0000000000
4	0.0000000000	0.0000000000	10.3628355130
5	-318.7583904805	-18.6065909522	0.0000000000
6	0.0000000000	0.0000000000	-54.1637848023
7	1393.0651368390	88.2305627304	0.0000000000
8	0.0000000000	0.0000000000	154.0605856734
9	-3483.7826186812	-233.5331157032	0.0000000000
10	0.0000000000	0.0000000000	-240.7596071527
11	4945.3954293408	345.2714224438	0.0000000000
12	0.0000000000	0.0000000000	194.7220465762
13	-3704.3036112783	-266.4104980060	0.0000000000
14	0.0000000000	0.0000000000	-63.7450060156
15	1136.1280526166	83.4342437049	0.0000000000

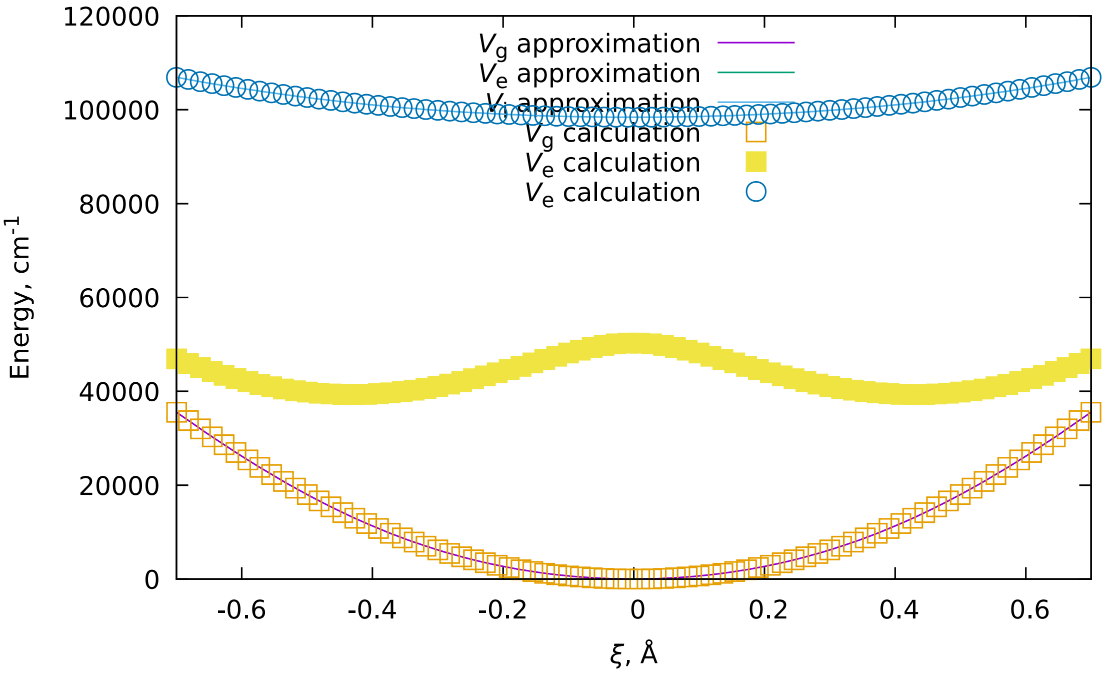

Figure S9 Potential energy surfaces of COFCl: Fitted ground and excited potential energy surfaces of the neutral COFCl ($V_{\rm g}(\xi)$ and $V_{\rm e}(\xi)$) and ground potential energy surface of the cationic COFCl ($V_{\rm i}(\xi)$) along the out-of-plane vibrational coordinate ξ. Points show the results of the quantum-chemical calculations, and solid lines illustrate the result of the fit.

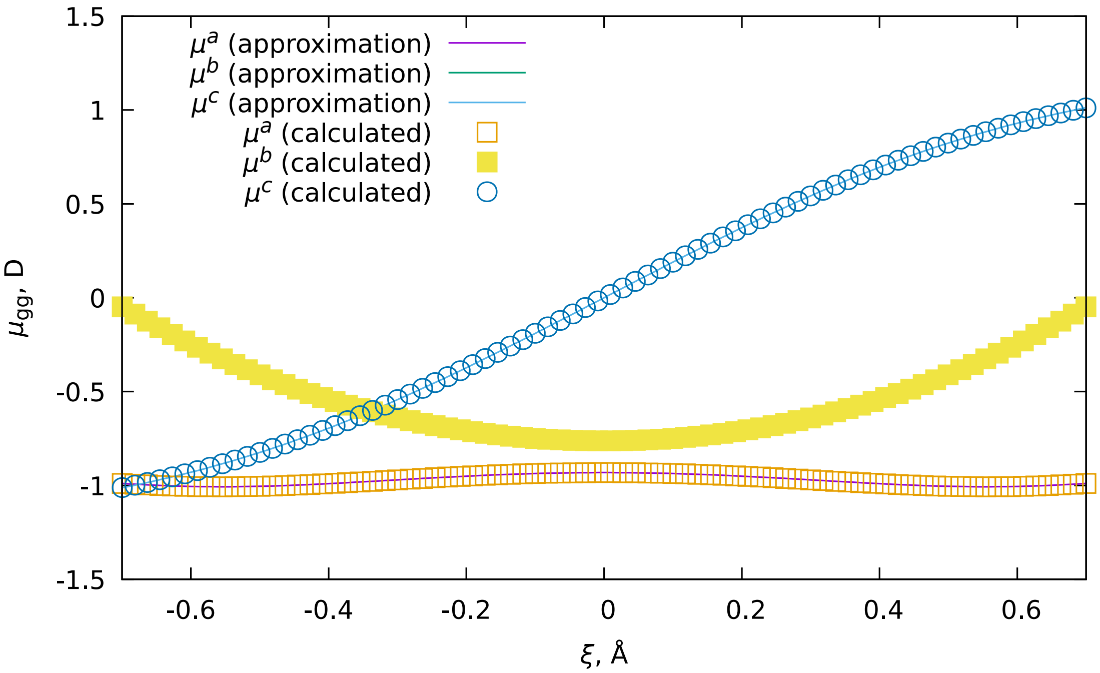

Figure S10 Dipole moments of COFCl: Fitted ground state dipole moments ($\mu^i_{\rm g}(\xi),\ i = a, b, c$) of the COFCl along the out-of-plane vibrational coordinate ξ. Points show the results of the quantum-chemical calculations, and solid lines illustrate the result of the fit.

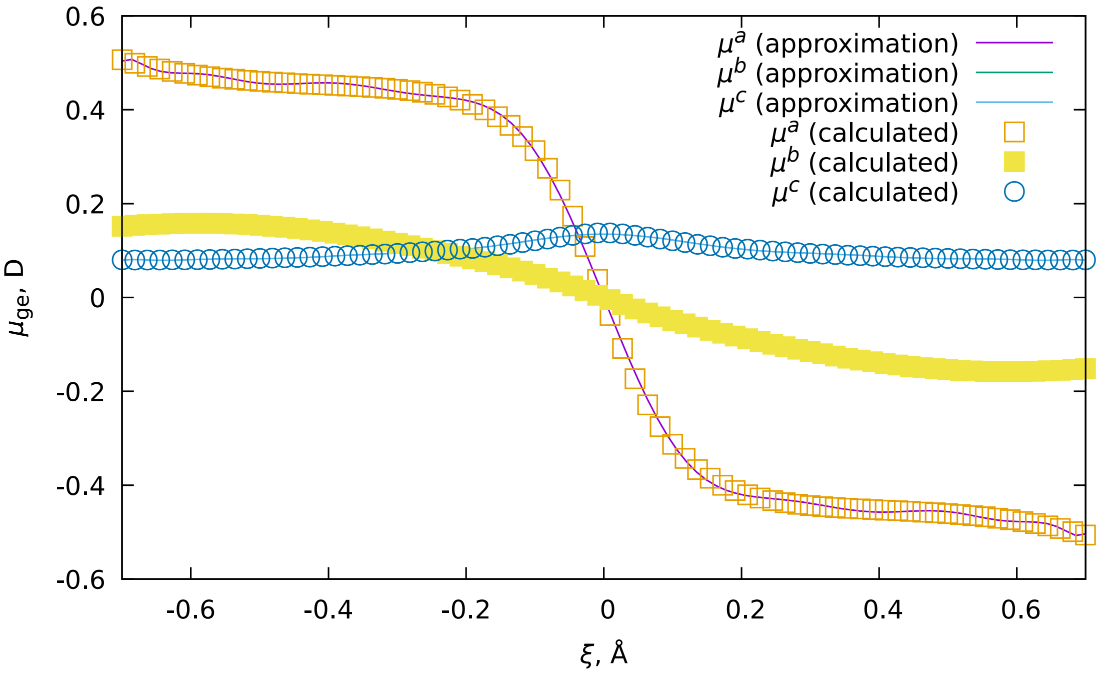

Figure S11 Transition dipole moments: Fitted ground to excited state transition dipole moments ($\mu^i_{\mathrm{g}}(\xi),\ i = a, b, c$) of the COFCl along the out-of-plane vibrational coordinate ξ. Points show the results of the quantum-chemical calculations, and solid lines illustrate the result of the fit.

V. CALCULATION OF THE CLASSICAL SELECTION RULES USING WXMAXIMA

Here, we provide the code in `wxMaxima` that was used to evaluate the selection rules for the enantiomeric signal, given in section I B.

```
R1(phi):= matrix([cos(phi), -sin(phi), 0],
                 [sin(phi),  cos(phi), 0],
                 [  0     ,    0,      1]);
R2(theta):= matrix([1, 0, 0],
                   [0, cos(theta), -sin(theta)],
                   [0, sin(theta),  cos(theta)]);
R3(chi) :=matrix([cos(chi), -sin(chi), 0],
                 [sin(chi),  cos(chi), 0],
                 [  0     ,    0,      1]);
R(phi,theta,chi) := ((R3(chi)).R2(theta)).(R1(phi));
J(theta):= sin(theta);

a1 : matrix([a1x,a1y,a1z]);
a2 : matrix([a2x,a2y,a2z]);
a3 : matrix([a3x,a3y,a3z]);
mu1 : matrix([m1x, m1y, m1z]);
mu2 : matrix([m2x, m2y, m2z]);
mu3 : matrix([m3x, m3y, m3z]);

Q1(phi,theta,chi):= (mu1.R(phi,theta,chi).a1);
integrate( integrate( integrate(Q1(phi,theta,chi)*J(theta), phi, 0, 2*\%pi ),
                                       theta, 0, \%pi )  , chi, 0 , 2*\%pi    );

Q2(phi,theta,chi):= Q1(phi,theta,chi)*(mu2.R(phi,theta,chi).a2);
integrate( integrate( integrate(Q2(phi,theta,chi)*J(theta), phi, 0, 2*\%pi ),
                                       theta, 0, \%pi )  , chi, 0 , 2*\%pi    );

Q3(phi,theta,chi):= Q2(phi,theta,chi)*(mu3.R(phi,theta,chi).a3);
integrate( integrate( integrate(Q3(phi,theta,chi)*J(theta), phi, 0, 2*\%pi ),
                                       theta, 0, \%pi )  , chi, 0 , 2*\%pi    );
```

VI. CALCULATION OF THE TRANSITION DIPOLE MOMENTS FROM THE ONE-DIMENSIONAL SCHRÖDINGER EQUATION

Here, we provide the `Python` code that was used for solving the one-dimensional Schrödinger equation to obtain the vibronic energy levels, the permanent dipole moments, and the transition dipole moments.

```python
import numpy as np
import sys
import argparse
import scipy.interpolate as spi
from copy import deepcopy
import scipy.linalg as spla

KinEnCoeff=33.7152313

def sincD1(i,j):
    if i==j:
        return 0.0
    else:
        return (-1.0)**(i-j)/(i-j)

def sincD2(i,j):
    if i==j:
        return -(np.pi**2)/3.0
```

```python
    else:
        return -2.0*(-1.0)**(i-j)/(i-j)**2


def KinEnergy(X,M):
    #G = 0.5*KinEnCoeff/M

    Nmat = len(X)
    dx = np.average(X[1:] - X[:-1])

    D2 = np.array([[sincD2(i,j) for j in range(0,Nmat)] for i in range(0,Nmat)])/(dx**2)

    print(" dx = %15.10f Angstroem" % dx)

    Res = -D2*0.5*KinEnCoeff/M

    print("Deviation from Hermitivity in T = %15.10f" % (np.max(np.abs( Res.flatten() - Res.T.flatten() ))) )
    return Res

def PotEnergy(V, Vmin=None):
    if Vmin is None:
        vmin = min(V)
    else:
        vmin=Vmin
    return np.diag(V-vmin)

########################################

parser = argparse.ArgumentParser()
parser.add_argument("-f", "--filename", help="input file name", type=str, default="ini.dat")
parser.add_argument("-m", "--mass", help="reduced mass of the vibration in a.m.u.", type=float, default=1.0)
parser.add_argument("--IniState", help="Initial state (>=0)", type=int, default=0)
parser.add_argument("--FinState", help="Final State Number (>=0)", type=int, default=1)
parser.add_argument("--NGS2P", help="Number of ground states to print", type=int, default=5)
parser.add_argument("--NES2P", help="Number of excated states to print", type=int, default=None)
parser.add_argument("-n", "--NumOfPts", help="Size of the Hamiltonian matrix", type=int, default=800)
parser.add_argument("--InterpolType", help="interpolation type", type=str, choices=['linear', 'quadratic', 'cubic'], default='cubic')
parser.add_argument('--SymmExc', action='store_true', help="Flag to symmetrize the excited state.")
parser.add_argument('--PrintPolTensors', action='store_true', help="Flag to print the polarizability tensors.")


args = parser.parse_args()



########################################
print("File to process: "+args.filename)
data = np.loadtxt(args.filename)

X0 = data[:,0]
Vgr = spi.interp1d(X0,data[:,1], kind=args.InterpolType)
Vex = spi.interp1d(X0,data[:,2], kind=args.InterpolType)

x = np.linspace(np.min(X0),np.max(X0),args.NumOfPts)



### Solve ground state problem
vg = Vgr(x)
ve = Vex(x)

Vmin = min(vg)

H = KinEnergy(x,args.mass) + PotEnergy(vg,Vmin=Vmin)
```

```
Eg,WFNg = np.linalg.eigh(H)

for n in range(0,len(Eg)):
    WFNg[:,n]/=np.sqrt(np.dot(WFNg[:,n],WFNg[:,n]))


print("Groung electronic state vibrational levels:\n")
for n in range(0,args.NGS2P):
    print(" %3i  %15.10f %15.10f " % (n, Eg[n], Eg[n]-Eg[0]) )

np.savetxt("dump0.wfn", np.stack([x] + [w for w in WFNg.T[0:args.NGS2P] ], axis=-1) )

Vmin = min(ve)
H = KinEnergy(x,args.mass) + PotEnergy(ve,Vmin=Vmin)

Ee,WFNe = np.linalg.eigh(H)

if args.SymmExc:
    print("Started symmetrization in the excited states, %i states in total" % len(Ee) )

    def symmWFN(wfn, Coeff=1.0):
        res = wfn + Coeff * np.flip(wfn)
        res /= np.sqrt(np.dot(res,res))
        return res

    # symmetric representation
    sWFNe = np.array([ symmWFN(wfn, Coeff=1.0) for wfn in WFNe.T ])

    rank = np.linalg.matrix_rank(sWFNe)
    print("Symmetric WFNs : %i" % rank)

    mask = True + np.zeros(sWFNe.shape[0], dtype=bool)
    print(mask[mask].shape)
    for i in range(1,sWFNe.shape[0]):
        for j in range(0,i):
            if np.abs(np.dot(sWFNe[i],sWFNe[j]))>0.5 and mask[j]:
                mask[i] = False
    print(mask[mask].shape)
    sWFNe = sWFNe[mask]
    np.savetxt("symmetric.wfn", np.concatenate([[x],sWFNe]).T   )

    sEe = np.array([np.dot(wfn,np.dot(H,wfn)) for wfn in sWFNe])

    # antisymmetric representation
    aWFNe = np.array([ symmWFN(wfn, Coeff=-1.0) for wfn in WFNe.T ])

    rank = np.linalg.matrix_rank(aWFNe)
    print("Antisymmetric WFNs : %i" % rank)

    mask = True + np.zeros(aWFNe.shape[0], dtype=bool)
    for i in range(1,aWFNe.shape[0]):
        for j in range(0,i):
            if np.abs(np.dot(aWFNe[i],aWFNe[j]))>0.5 and mask[j]:
                mask[i] = False
    print(mask[mask].shape)
    aWFNe = aWFNe[mask]
    np.savetxt("antisymmetric.wfn", np.concatenate([[x],aWFNe]).T   )

    aEe = np.array([np.dot(wfn,np.dot(H,wfn)) for wfn in aWFNe])

    WFNe = np.concatenate([sWFNe,aWFNe])
    Ee = np.concatenate([sEe,aEe])
    print(WFNe.shape,Ee.shape)

    inds = np.argsort(Ee)

    WFNe = WFNe[inds].T
    Ee   = Ee[inds]
    print(WFNe.shape,Ee.shape)

```

```
if args.NES2P is None:
    args.NES2P = len(Ee)


Ee += min(ve) - min(vg)


for n in range(0,len(Ee)):
    WFNe[:,n]/=np.sqrt(np.dot(WFNe[:,n],WFNe[:,n]))


print("Excited electronic state vibrational levels:\n")
for n in range(0,args.NES2P):
    print(" %3i  %15.10f %15.10f  %15.10f" % (n, Ee[n], Ee[n]-Ee[0], Ee[n]-Eg[0]) )

np.savetxt("dump1.wfn", np.stack([x] + [w for w in WFNe.T[0:args.NES2P] ], axis=-1) )

TrD=None
if len(data[0])>=6:
    TrD=[]
    TrD.append(spi.interp1d(X0,data[:,3], kind=args.InterpolType)(x)  )
    TrD.append(spi.interp1d(X0,data[:,4], kind=args.InterpolType)(x)  )
    TrD.append(spi.interp1d(X0,data[:,5], kind=args.InterpolType)(x)  )


D0=None
if len(data[0])>=9:
    D0=[]
    D0.append(spi.interp1d(X0,data[:,6], kind=args.InterpolType)(x)  )
    D0.append(spi.interp1d(X0,data[:,7], kind=args.InterpolType)(x)  )
    D0.append(spi.interp1d(X0,data[:,8], kind=args.InterpolType)(x)  )


np.set_printoptions(suppress=True, precision=12)


outf  = open("raman_and_static.res","w")
outf1 = open("absorbtion.sp","w")

if args.PrintPolTensors:
    outf2 = open("pol_tensors.dat", "w")


d00 = np.array([np.dot(WFNg[:,args.IniState],D0[i]*WFNg[:,args.IniState]) for i in range(0,3) ])
print(d00)


for n,vState in enumerate(WFNe.T):

    E0v = Ee[n]-Eg[args.IniState]
    Ev1 = Ee[n]-Eg[args.FinState]

    d0n = np.array([np.dot(vState,TrD[i]*WFNg[:,args.IniState]) for i in range(0,3) ])
    dn1 = np.array([np.dot(vState,TrD[i]*WFNg[:,args.FinState]) for i in range(0,3) ])


    dV = np.dot(d00, np.cross(d0n,dn1))
    outf.write("  %15.2f  %15.2f   %15.10f   " % (E0v, Ev1, dV) )
    outf.write("\n")

    outf1.write("  %15.3f  %15.3f %15.10f  \n" % (E0v, 1.0e7/E0v, np.dot(d0n,d0n)) )

    if args.PrintPolTensors:
        outf2.write("\n\n |%i,g> -> |%i,e> -> |%i,g> : \n" % (args.IniState, n, args.FinState) )
        pt = np.outer(d0n,dn1)
        outf2.write(str(pt)+"\n\n")

D2S = [x,vg,ve]
if not TrD is None:
```

```
    D2S = D2S + TrD
if not D0 is None:
    D2S = D2S + D0

np.savetxt("extrapolated_values.dat", np.stack(D2S ,axis=-1))
```

REFERENCES AND NOTES

1. D. P. Glavin, A. S. Burton, J. E. Elsila, J. C. Aponte, J. P. Dworkin, The search for chiral asymmetry as a potential biosignature in our solar system. *Chem. Rev.* **120**, 4660–4689 (2020).
2. M. Quack, J. Stohner, M. Willeke, High-resolution spectroscopic studies and theory of parity violation in chiral molecules. *Annu. Rev. Phys. Chem.* **59**, 741–769 (2008).
3. U. Raucci, H. Weir, C. Bannwarth, D. M. Sanchez, T. J. Martínez, Chiral photochemistry of achiral molecules. *Nat. Commun.* **13**, 2091 (2022).
4. C. Fábri, L. Horný, M. Quack, Tunneling and parity violation in trisulfane (HSSSH): An almost ideal molecule for detecting parity violation in chiral molecules. *ChemPhysChem* **16**, 3584–3589 (2015).
5. Y. Fujimura, L. González, K. Hoki, J. Manz, Y. Ohtsuki, Selective preparation of enantiomers by laser pulses: Quantum model simulation for H_2POSH. *Chem. Phys. Lett.* **306**, 1–8 (1999).
6. D. Gerbasi, P. Brumer, I. Thanopulos, P. Král, M. Shapiro, Theory of the two step enantiomeric purification of 1,3 dimethylallene. *J. Chem. Phys.* **120**, 11557–11563 (2004).
7. E. F. Thomas, N. E. Henriksen, Phase-modulated nonresonant laser pulses can selectively convert enantiomers in a racemic mixture. *J. Phys. Chem. Lett.* **8**, 2212–2219 (2017).
8. M. Quack, G. Seyfang, G. Wichmann, Fundamental and approximate symmetries, parity violation and tunneling in chiral and achiral molecules. *Adv. Quantum Chem.* **81**, 51–104 (2020).
9. A. F. Ordonez, O. Smirnova, Generalized perspective on chiral measurements without magnetic interactions. *Phys. Rev. A* **98**, 063428 (2018).
10. B. Ritchie, Theory of the angular distribution of photoelectrons ejected from optically active molecules and molecular negative ions. *Phys. Rev. A* **13**, 1411–1415 (1976).
11. N. Böwering, T. Lischke, B. Schmidtke, N. Müller, T. Khalil, U. Heinzmann, Asymmetry in photoelectron emission from chiral molecules induced by circularly polarized light. *Phys. Rev. Lett.* **86**, 1187–1190 (2001).
12. C. Lux, M. Wollenhaupt, T. Bolze, Q. Liang, J. Köhler, C. Sarpe, T. Baumert, Circular dichroism in the photoelectron angular distributions of camphor and fenchone from multiphoton ionization with femtosecond laser pulses. *Angew. Chem. Int. Ed.* **51**, 5001–5005 (2012).
13. A. Comby, S. Beaulieu, M. Boggio-Pasqua, D. Descamps, F. Légaré, L. Nahon, S. Petit, B. Pons, B. Fabre, Y. Mairesse, V. Blanchet, Relaxation dynamics in photoexcited chiral molecules studied by

time-resolved photoelectron circular dichroism: Toward chiral femtochemistry. *J. Phys. Chem. Lett.* **7**, 4514–4519 (2016).

14. S. Beaulieu, A. Comby, A. Clergerie, J. Caillat, D. Descamps, N. Dudovich, B. Fabre, R. Géneaux, F. Légaré, S. Petit, B. Pons, G. Porat, T. Ruchon, R. Taïeb, V. Blanchet, Y. Mairesse, Attosecond-resolved photoionization of chiral molecules. *Science* **358**, 1288–1294 (2017).
15. V. Svoboda, N. B. Ram, D. Baykusheva, D. Zindel, M. D. J. Waters, B. Spenger, M. Ochsner, H. Herburger, J. Stohner, H. J. Wörner, Femtosecond photoelectron circular dichroism of chemical reactions. arXiv:2206.04099 [physics.chem-ph] (8 June 2022).
16. K. Fehre, S. Eckart, M. Kunitski, M. Pitzer, S. Zeller, C. Janke, D. Trabert, J. Rist, M. Weller, A. Hartung, L. P. H. Schmidt, T. Jahnke, R. Berger, R. Dörner, M. S. Schöffler, Enantioselective fragmentation of an achiral molecule in a strong laser field. *Sci. Adv.* **5**, eaau7923 (2019).
17. D. Baykusheva, D. Zindel, V. Svoboda, E. Bommeli, M. Ochsner, A. Tehlar, H. J. Wörner, Real-time probing of chirality during a chemical reaction. *Proc. Natl. Acad. Sci. U.S.A.* **116**, 23923–23929 (2019).
18. S. S. Bychkov, B. A. Grishanin, V. N. Zadkov, H. Takahashi, Laser coherent control of molecular chiral states via entanglement of the rotational and torsional degrees of freedom. *J. Raman Spectrosc.* **33**, 962–973 (2002).
19. D. V. Zhdanov, V. N. Zadkov, Coherent control of chirality in ensemble of randomly oriented molecules using a sequence of short laser pulses. *Laser Physics* **20**, 107–118 (2010).
20. A. F. Ordonez, O. Smirnova, Propensity rules in photoelectron circular dichroism in chiral molecules. I. Chiral hydrogen. *Phys. Rev. A* **99**, 043416 (2019).
21. C. P. Koch, M. Lemeshko, D. Sugny, Quantum control of molecular rotation. *Rev. Mod. Phys.* **91**, 035005 (2019).
22. D. L. Andrews, T. Thirunamachandran, On three-dimensional rotational averages. *J. Chem. Phys.* **67**, 5026–5033 (1977).
23. M. Leibscher, T. F. Giesen, C. P. Koch, Principles of enantio-selective excitation in three-wave mixing spectroscopy of chiral molecules. *J. Chem. Phys.* **151**, 014302 (2019).
24. I. Powis, in *Photoelectron Circular Dichroism in Chiral Molecules*, S. A. Rice, Ed. (John Wiley & Sons Ltd., 2008), chap. 5, pp. 267–329.
25. M. H. M. Janssen, I. Powis, Detecting chirality in molecules by imaging photoelectron circular dichroism. *Phys. Chem. Chem. Phys.* **16**, 856–871 (2014).

26. C. Lux, M. Wollenhaupt, C. Sarpe, T. Baumert, Photoelectron circular dichroism of bicyclic ketones from multiphoton ionization with femtosecond laser pulses. *ChemPhysChem* **16**, 115–137 (2015).
27. A. Kastner, C. Lux, T. Ring, S. Züllighoven, C. Sarpe, A. Senftleben, T. Baumert, Enantiomeric excess sensitivity to below one percent by using femtosecond photoelectron circular dichroism. *ChemPhysChem* **17**, 1119–1122 (2016).
28. G. Meijer, in *Molecular Beams in Physics and Chemistry: From Otto Stern's Pioneering Exploits to Present-Day Feats*, B. Friedrich, H. Schmidt-Böcking, Eds. (Springer International Publishing, 2021), pp. 463–476.
29. R. E. Goetz, C. P. Koch, L. Greenman, Quantum control of photoelectron circular dichroism. *Phys. Rev. Lett.* **122**, 013204 (2019).
30. R. E. Goetz, C. P. Koch, L. Greenman, Perfect control of photoelectron anisotropy for randomly oriented ensembles of molecules by XUV REMPI and polarization shaping. *J. Chem. Phys.* **151**, 074106 (2019).
31. E. Ruch, Chiral derivatives of achiral molecules: Standard classes and the problem of a right-left classification. *Angew. Chem. Int. Ed.* **16**, 65–72 (1977).
32. G. Hartmann, M. Ilchen, P. Schmidt, C. Küstner-Wetekam, C. Ozga, F. Scholz, J. Buck, F. Trinter, J. Viefhaus, A. Ehresmann, M. S. Schöffler, A. Knie, P. V. Demekhin, Recovery of high-energy photoelectron circular dichroism through fano interference. *Phys. Rev. Lett.* **123**, 043202 (2019).
33. B. Friedrich, D. Herschbach, Enhanced orientation of polar molecules by combined electrostatic and nonresonant induced dipole forces. *J. Chem. Phys.* **111**, 6157–6160 (1999).
34. S. V. Krasnoshchekov, V. B. Laptev, S. A. Klimin, I. K. Gainullin, A. A. Makarov, Overtone spectroscopy of ν(C=O) stretching vibration of hexafluoroacetone: Experimental and ab initio determination of peak positions, absolute intensities, and band shapes. *Spectrochim. Acta A Mol. Biomol. Spectrosc.* **238**, 118396 (2020).
35. S. Takeda, R. Tarao, The infrared spectra of alkylaluminum-ether complexes. *Bull. Chem. Soc. Jpn.* **38**, 1567–1574 (1965).
36. H. Hoberg, S. Krause, The Al—Al bond as a readily accessible structural unit in organometallic compounds. *Angew. Chem. Int. Ed.* **15**, 694–694 (1976).
37. A. Kunicki, W. Kosińska, M. Bolesławski, S. Pasynkiewicz, The 1H NMR spectra of methylmethoxyaluminium chloride and methylmethoxyaluminium iodide. *J. Organomet. Chem.* **141**, 283–288 (1977).

38. A. D. Becke, Density-functional thermochemistry. III. The role of exact exchange. *J. Chem. Phys.* **98**, 5648–5652 (1993).
39. C. Lee, W. Yang, R. G. Parr, Development of the Colle-Salvetti correlation-energy formula into a functional of the electron density. *Phys. Rev. B* **37**, 785–789 (1988).
40. S. H. Vosko, L. Wilk, M. Nusair, Accurate spin-dependent electron liquid correlation energies for local spin density calculations: A critical analysis. *Can. J. Phys.* **59**, 1200–1211 (1980).
41. P. J. Stephens, F. J. Devlin, C. F. Chabalowski, M. J. Frisch, Ab initio calculation of vibrational absorption and circular dichroism spectra using density functional force fields. *J. Phys. Chem.* **98**, 11623–11627 (1994).
42. T. H. Dunning Jr., Gaussian basis sets for use in correlated molecular calculations. i. The atoms boron through neon and hydrogen. *J. Chem. Phys.* **90**, 1007–1023 (1989).
43. S. Hirata, M. Head-Gordon, Time-dependent density functional theory within the Tamm-Dancoff approximation. *Chem. Phys. Lett.* **314**, 291–299 (1999).
44. F. Neese, The ORCA program system. *Wiley Interdiscip. Rev. Comput. Mol. Sci.* **2**, 73–78 (2012).
45. D. T. Colbert, W. H. Miller, A novel discrete variable representation for quantum mechanical reactive scattering via the *S*-matrix Kohn method. *J. Chem. Phys.* **96**, 1982–1991 (1992).
46. I. Zanon, G. Giacometti, D. Picciol, The electronic spectrum of FCICO. *Spectrochim. Acta A Mol. Biomol. Spectrosc.* **19**, 301-E7 (1963).
47. N. Heineking, W. Jager, M. Gerry, Isotopic and vibrational satellites in the rotational spectrum of carbonyl chloride fluoride. *J. Mol. Spectrosc.* **158**, 69–81 (1993).
48. C. Maul, C. Dietrich, T. Haas, K.-H. Gericke, Photodissociation dynamics of carbonyl chloride fluoride and its implications for phosgene three body decay. *Phys. Chem. Chem. Phys.* **1**, 1441–1446 (1999).
49. A. Perrin, J. Demaison, G. Toon, The ν_1, ν_2, and ν_3 bands of carbonyl chlorofluoride (COFCl) at 5.3, 9.1, and 13.1 µm: Position and intensity parameters and their use for atmospheric studies. *J. Quant. Spectrosc. Radiat. Transf.* **112**, 1266–1279 (2011).
50. H. Keller-Rudek, G. K. Moortgat, R. Sander, R. Sörensen, The MPI-Mainz UV/VIS spectral atlas of gaseous molecules of atmospheric interest. *Earth Syst. Sci. Data* **5**, 365–373 (2013).
51. H.-J. Werner, P. J. Knowles, G. Knizia, F. R. Manby, M. Schütz, P. Celani, T. Korona, R. Lindh, A. Mitrushenkov, G. Rauhut, K. R. Shamasundar, T. B. Adler, R. D. Amos, A. Bernhardsson, A. Berning, D. L. Cooper, M. J. O. Deegan, A. J. Dobbyn, F. Eckert, E. Goll, C. H. l, A. Hesselmann, G.

Hetzer, T. Hrenar, G. Jansen, C. Köppl, Y. Liu, A. W. Lloyd, R. A. Mata, A. J. May, S. J. McNicholas, W. Meyer, M. E. Mura, A. Nicklass, D. P. O'Neill, P. Palmieri, D. Peng, K. Pflüger, R. Pitzer, M. Reiher, T. Shiozaki, H. Stoll, A. J. Stone, R. Tarroni, T. Thorsteinsson, M. Wang, MOLPRO, version 2012.1, a package of ab initio programs.

52. H.-J. Werner, P. J. Knowles, G. Knizia, F. R. Manby, M. Schütz, Molpro: A general-purpose quantum chemistry program package. *Wiley Interdiscip. Rev. Comput. Mol. Sci.* **2**, 242–253 (2012).
53. R. A. Kendall, T. H. Dunning Jr., R. J. Harrison, Electron affinities of the first-row atoms revisited. Systematic basis sets and wave functions. *J. Comp. Phys.* **96**, 6796–6806 (1992).
54. F. A. Gianturco, R. R. Lucchese, N. Sanna, Calculation of low-energy elastic cross sections for electron-CF_4 scattering. *J. Chem. Phys.* **100**, 6464–6471 (1994).
55. A. P. P. Natalense, R. R. Lucchese, Cross section and asymmetry parameter calculation for sulfur 1s photoionization of SF6. *J. Chem. Phys.* **111**, 5344–5348 (1999).
56. P. Hockett, M. Wollenhaupt, C. Lux, T. Baumert, Complete photoionization experiments via ultrafast coherent control with polarization multiplexing. II. Numerics and analysis methodologies. *Phys. Rev. A* **92**, 013411 (2015).
57. S. Grimme, S. Ehrlich, L. Goerigk, Effect of the damping function in dispersion corrected density functional theory. *J. Comput. Chem.* **32**, 1456–1465 (2011).
58. F. Weigend, R. Ahlrichs, Balanced basis sets of split valence, triple zeta valence and quadruple zeta valence quality for h to rn: Design and assessment of accuracy. *Phys. Chem. Chem. Phys.* **7**, 3297–3305 (2005).
59. P. Atkins, J. Paula, *Atkins' Physical Chemistry* (Oxford Univ. Press, 2008).
60. M. Mantina, A. C. Chamberlin, R. Valero, C. J. Cramer, D. G. Truhlar, Consistent van der Waals radii for the whole main group. *Chem. A Eur. J.* **113**, 5806–5812 (2009).
